# Review: Electrostatically actuated nanobeam-based nanoelectromechanical switches – materials solutions and operational conditions

**DOI:** 10.3762/bjnano.9.29

**Published:** 2018-01-25

**Authors:** Liga Jasulaneca, Jelena Kosmaca, Raimonds Meija, Jana Andzane, Donats Erts

**Affiliations:** 1 Institute of Chemical Physics; 2 Department of Chemistry, University of Latvia, Raina Blvd. 19, Riga, LV-1586, Latvia

**Keywords:** nanocontacts, nanoelectromechanical switches, nanowires, NEM, reliability

## Abstract

This review summarizes relevant research in the field of electrostatically actuated nanobeam-based nanoelectromechanical (NEM) switches. The main switch architectures and structural elements are briefly described and compared. Investigation methods that allow for exploring coupled electromechanical interactions as well as studies of mechanically or electrically induced effects are covered. An examination of the complex nanocontact behaviour during various stages of the switching cycle is provided. The choice of the switching element and the electrode is addressed from the materials perspective, detailing the benefits and drawbacks for each. An overview of experimentally demonstrated NEM switching devices is provided, and together with their operational parameters, the reliability issues and impact of the operating environment are discussed. Finally, the most common NEM switch failure modes and the physical mechanisms behind them are reviewed and solutions proposed.

## Review

### Introduction

Nanoelectromechanical (NEM) switches represent a class of nanoscale devices, integrating both electrical and mechanical functionality of nanostructures to process external stimuli applied to the device and controlling the electrical current. NEM switches have attracted attention as low-power [1] devices, demonstrating abrupt on/off switching characteristics and minimized sub-threshold swing, as well as reduced leakage currents leading to improved on/off ratios [2]. In the context of existing microelectromechanical (MEM) switches, downsizing to the nanoscale leads to lower power consumption, increased switching speed, integration density and higher precision. While the characteristic dimensions of MEM switch components are between 1 µm and 1 mm, for the NEM scale devices, it is below 100 nm. This means that NEM switches combine the advantages of a smaller mass with a higher surface area to volume ratio. When the linear dimension scale decreases by three orders of magnitude, the area and volume decrease by six and nine orders of magnitude, respectively. Surface forces, proportional to area, become a thousand times larger than forces that are proportional to volume, making inertial forces negligible. At the nanoscale, van der Waals, capillary and electrostatic forces become the governing factors. It is important to note, that with the decrease of the size scale, breakdown of the predictions of continuum-based theories may occur. For instance, for cantilevered resonating nanostructures, continuum mechanics predictions fail when the cross-sectional area of the nanostructure is on the order of tens of lattice constants [3]. At this level, quantum effects, crystalline perfection, surface and interface interactions govern materials properties and behaviour [4]. Thus, NEM switches provide an exciting opportunity for gaining fundamental insight in such fields as surface science and electrical and mechanical processes in nanocontacts.

The NEM switch components can be produced from a wide range of nanostructures (e.g., thin films [5–7], nanobundles [8], nanowires [9–14], nanotubes [15–16]) fabricated from different materials (e.g., metals [17–19], semiconductors [9–10,20–23], carbon allotropes, including graphene [24–31] and carbon nanotubes [12,15–16,32–37]). With a proper choice of material and architecture, NEM switches can withstand relatively high radiation levels and extreme temperatures [19,25,38–39], highlighting their potential for applications in harsh environments.

Currently, the investigation of NEM switches is mostly focused on developing experimental approaches for device prototype fabrication and testing in laboratory environment and theoretical modelling based on continuum mechanics and molecular dynamics, allowing simulations to be performed on the processes occurring in NEM switching devices and the analysis of their working parameters [40–46]. A common approach for experimental investigation on NEM switches includes device fabrication and testing on chip. The fabrication can be based either on entirely top-down, or on a combination of top-down, bottom-up and nanomanipulation approaches. The top-down approach involves lithography, etching and coating technologies to fabricate device structures from bulk materials or thin films [7,19,23,39,47–50]. The combined approach of fabricating NEM switches requires subsequent transfer and alignment of synthesized nanostructures (nanowires, nanotubes, nanorods, graphene) with a good uniformity and desired properties. The microfabrication routine may be supplemented with some bottom-up approaches. Dielectrophoresis [33], controlled nanomaterial growth [34], and nanomanipulation [9] have been demonstrated as useful methods for small batch device fabrication with future prospects for scalable production of NEM switches on chip.

Another approach for fabrication and characterization of NEM device prototypes is in situ measurement technique, where the switch elements are positioned using nanomanipulators inside an electron microscope [8,10–15]. In situ studies of the dynamics of force interactions, conductance and adhesion in gold point contacts using combined transmission electron microscopy/scanning tunnelling microscopy (TEM-STM) [51–52] and atomic force microscopy/transmission electron microscopy (AFM-TEM) [53] showed suitability of these techniques for further NEM switch related research. A major advantage of in situ experiments is that the device geometry and operation can be visualized and adjusted in real time within one experimental session. This allows optimization of the geometry of a NEM device, avoiding the need for individual device fabrication of each control parameter. This approach is favourable for fundamental research on specific parts and simulation of processes in a NEM switch, where the assembly of the whole device is not required. Real-time visualization helps to evaluate the contact area [8,10–11,54], observe the electrical breakdown mechanism for a single nanostructure [55–58], and investigate the dynamic processes occurring in the switch nanocontact [54].

The durability of a NEM switch strongly depends on the evolution of the contact between the switching element and the contact electrode during NEM switch operation. The increase of adhesion in the contact or its conductivity reduction down to the noise level with repetitive switching degrades the device stability and often leads to device failure. The state of the art lifetime of NEM switches varies from one-off laboratory-scale measurements [59], demonstrating a few tens [10–11,26,47,49,60] of switching cycles, up to devices showing 10^4^–10^8^ [12,18–19,38–39,61] switching cycles. However, to achieve a technology readiness level suitable for commercial applications, NEM switches should endure up to 10^15^ cycles without failing [59,62–63]. Thus, the reliability of NEM switches is one of the most critical issues that slow down wider adoption of this technology. Further efforts are necessary to select the material combination and suitable architecture that meet the requirements for commercial applications.

Actuation of NEM switches includes a variety of methods, for example, electrostatic [7,12], thermal [64], piezoelectric [65], resonant [66] and free-floating [67] switching. Electrostatic actuation is one of the most widespread and actively studied actuation modes. It is a promising method for operating nanosized switches due to its simple process requirements. More advantages of the electrostatically actuated NEM contact switches are temperature-independent actuation characteristics and reduction in power consumption with scaling. The device configurations where electrostatically actuated single- or double-clamped nanobeams are used as switching elements have great potential for architecture modification of the device and are widely used in development of NEM contact switch prototypes. The aim of this work is to review recent research carried out on electrostatically actuated nanobeam-based NEM contact switches, fabricated by different methods. The processes occurring in nanocontacts and the improvement of their reliability in terms of choice of materials for both electrodes and switching elements are covered in the light of the experimental findings in the field. Methods and approaches for investigating the behaviour of NEM switches as well as environmental considerations and failure modes are discussed. This review does not cover research into the use of other types of NEM switches based on, for example, free-floating [67] or resonant switching [66] principles, as well as thermal [64] or piezoelectric [65] actuation methods.

### Main architectures and basic operational principles of electrostatically actuated nanobeam-based NEM switches

In general, electrostatically actuated nanobeam-based NEM switches can be divided into two main groups: two-terminal (2T) switches, employing only source and drain electrodes, and three-terminal (3T) and more terminal switches, employing an additional single or multiple gate electrodes in single-clamped and double-clamped configurations.

#### Two-terminal NEM switches

**Figure 1:**
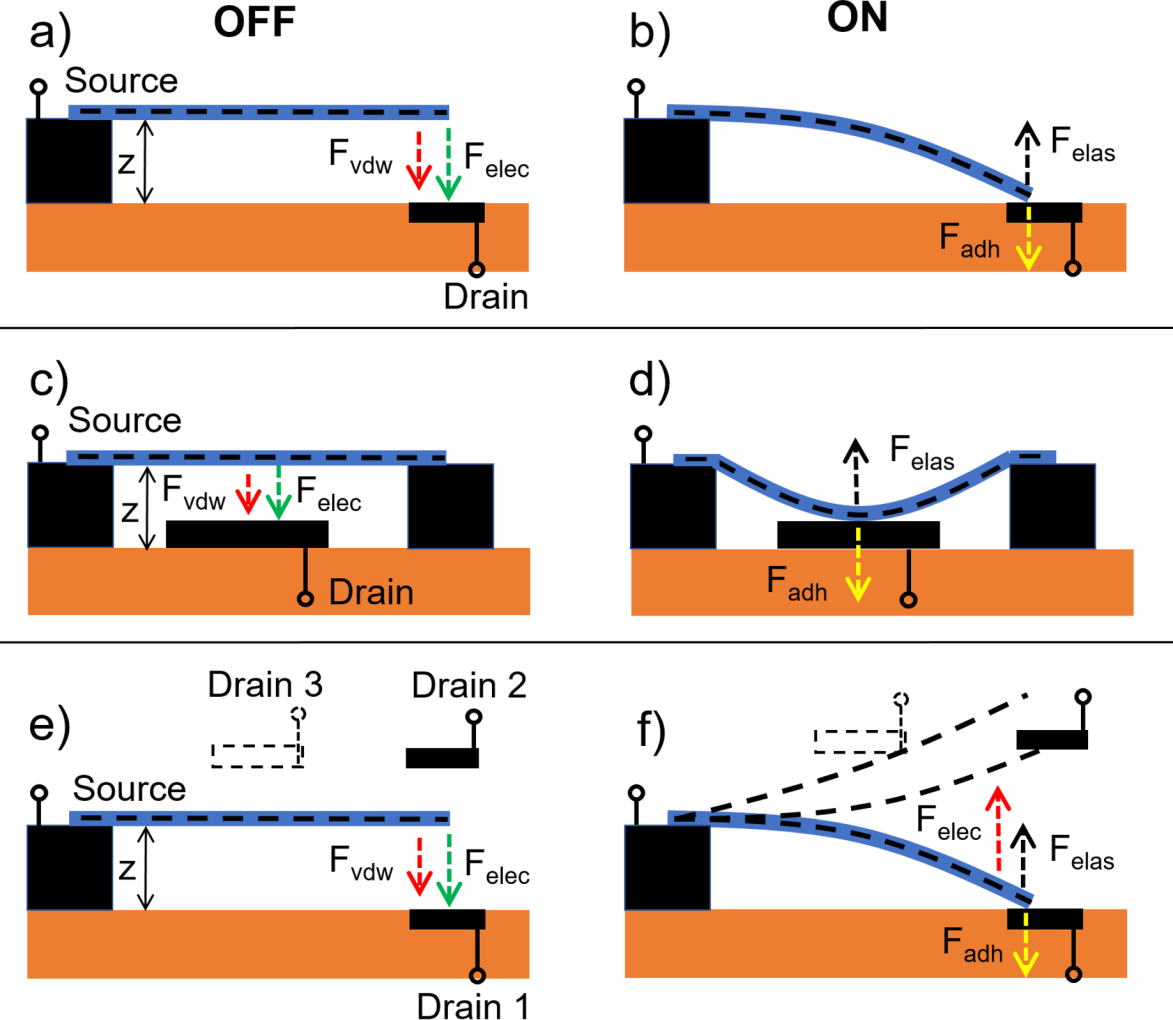
Schematics of electrostatically actuated 2T NEM nanobeam-based switches. Top panel: single-clamped 2T NEM switch in a) off and b) on state. Middle panel: double-clamped 2T NEM switch in c) off and d) on state. Bottom panel: single-clamped NEM switch with multiple drain electrodes in e) off and f) on state.

The full NEM switching cycle consists of establishment of a mechanical and electrical contact between a switching element and a contact electrode, which is followed by electrical-current-assisted processes in the on state, and a subsequent disengagement of both electrical and mechanical contacts while switching back to the off state. The switching cycle is based on balancing attractive (van der Waals (*F*_vdW_) and electrostatic (*F*_elec_)) and repulsive elastic (*F*_elas_) forces acting on a movable switching element in single (Figure 1a) or double-clamped (Figure 1c) position, and initially separated from the contact electrode by a distance *z*.

**Figure 2:**
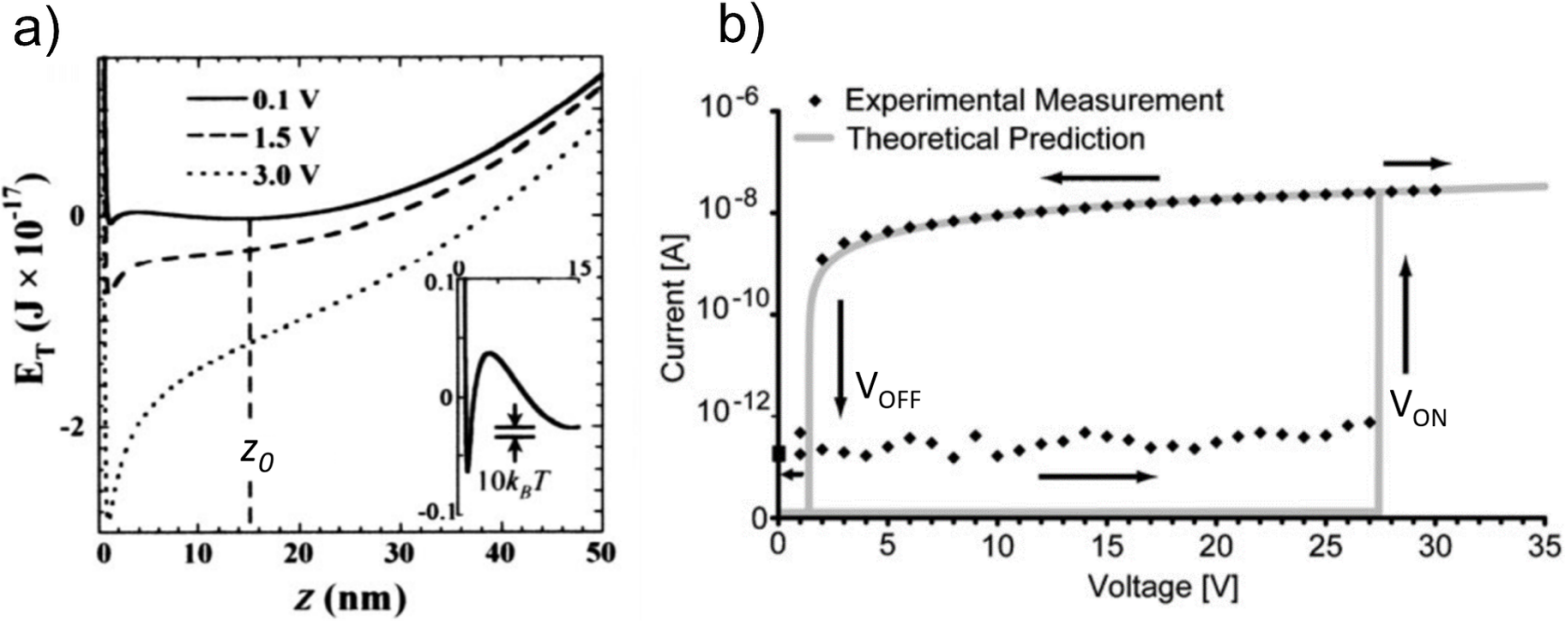
a) Plots of total energy *E*_T_ calculated for a Ge nanowire-based NEM device at different electrostatic potentials. The electrode surface is located at *z* = 0. Inset shows the energy barrier between the two stable (on/off) minima in relation to 10*k*_B_*T*. Reprinted with permission from [13], copyright 2004 AIPP. b) Comparison between experimentally measured (diamonds) and theoretically predicted (solid line) switching cycles for a carbon nanotube-based 2T bistable NEM switch, showing a sharp transition to the on state and a typical hysteresis behaviour. Reprinted with permission from [15], copyright 2006 John Wiley & Sons, Inc.

The operation principle of a NEM switch can be described from the viewpoint of potential energy *E* = ∫ *F* d*z* of the switching element, where *F* is the force acting on the switching element and *z* is the separation distance between the switching element and the contact electrode. Its total potential energy can be expressed as follows:

[1]
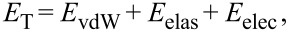


where *E*_vdW_ is the van der Waals (vdW) energy, *E*_elas_ is the elastic energy and *E*_elec_ is the electrostatic energy. An example of potential energy diagrams for the switching element–electrode interactions is shown in Figure 2a [13]. At low or no electrostatic field applied between the switching element and electrode there are two local minima located respectively at an initial switching element/electrode separation distance *z*_0_, and at a few nanometres from the electrode surface *z*_vdW_. The minimum located close to the electrode surface (at distance *z*_vdW_*)* is related to attractive vdW forces. The minimum located at *z*_0_ is related to the off state of the NEM switch, where the elastic energy of switching element that is freely suspended over the electrode is minimal (Figure 1a, c; Figure 2a solid line). For *z*_0_* >> z*_vdW_*,* the *E*_T_(*z*) curve would be symmetric with respect to the minimum located at *z*_0_. However, at close distances between the switching element and the electrode surface, the vdW interaction energy sufficiently reduces the total potential energy. Applying an increasingly larger electrostatic force diminishes the potential barrier until its complete elimination (Figure 2a dashed and dotted lines) and results in deflection of the switching element towards the contact electrode. For on and off states of a NEM switch to remain stable in the on state at room temperature, the potential barrier between energy minima must be much larger than 10*k*_B_*T* (*k*_B_ is the Boltzmann constant and *T* is temperature) (Figure 2a, inset) [32].

When the gradient of total attractive force exceeds the spring constant of the switching element approaching the electrode surface, the switching element starts accelerating. This is followed by establishment of mechanical and electrical contact between them (jump-in), and consequently, initiation of a current flow in the circuit (Figure 1b,d; Figure 2b) [13,15,32,40,68].

The jump-in voltage depends on the geometry of the device and the stiffness of the switching element. Switching from the on to off state (jump-off) occurs when the spring constant of the switching element exceeds the gradient of the total attractive force *F*_adh_ at the contact between the switching element and the electrode, and can be seen in *I*(*V*) curves as a sudden decrease of electrical current down to the noise level (Figure 2b).

Since operation of NEM switches is substantially determined by adhesion forces due to significant contribution of vdW forces [69], jump-off occurs at lower voltages in comparison to jump-in, and typical hysteresis loops in *I*(*V*) curves of NEM switches are observed [8,10,15]. A hysteresis loop is illustrated in Figure 2b showing experimentally obtained results [15] in comparison with the results of calculations assuming that the switching element is a uniform linear elastic beam and a perfect conductor [41–42].

**Figure 3:**
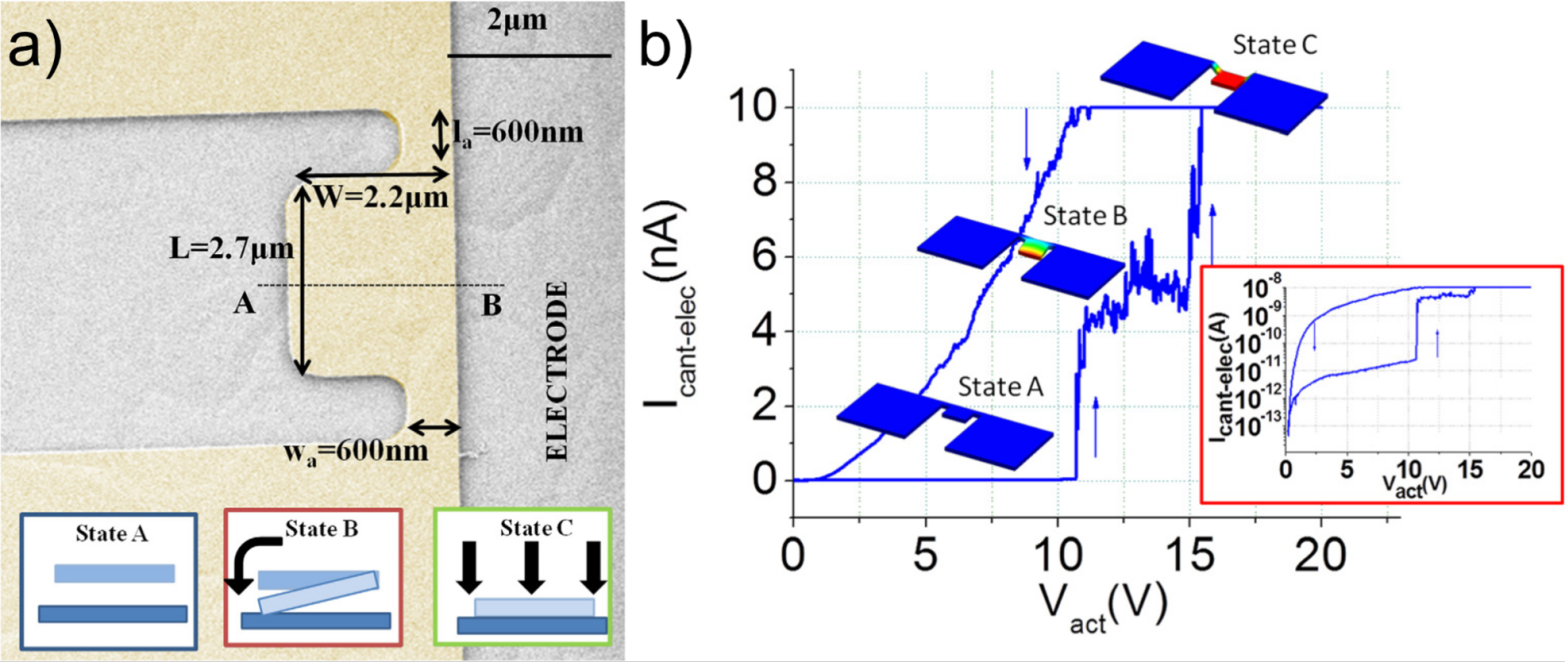
Operation of a 2T switch with a semi-paddle configuration. a) Image of the fabricated switch and schematic representation of its operational states and b) switching characteristics of the device, showing three operation states A (off), B (torsional movement) and C (flexural movement). Reprinted with permission from [48], copyright 2014 AIPP.

To expand the functionality of the 2T configuration switch, specially designed semi-paddle structures, allowing torsional movement of the switching element and consequently three different operation states can be used, as it was shown for a 2T TiN NEM switch [48] (Figure 3).

The off state is established when the switching element is separated from the contact electrode (Figure 3, state A), and two successive on states with different pull-in voltages (Figure 3, states B and C) can be established by making contact between the switching element and the electrode due to either torsional or flexural movement of the paddle anchors. Such architecture may serve for memory and logic applications.

**Figure 4:**
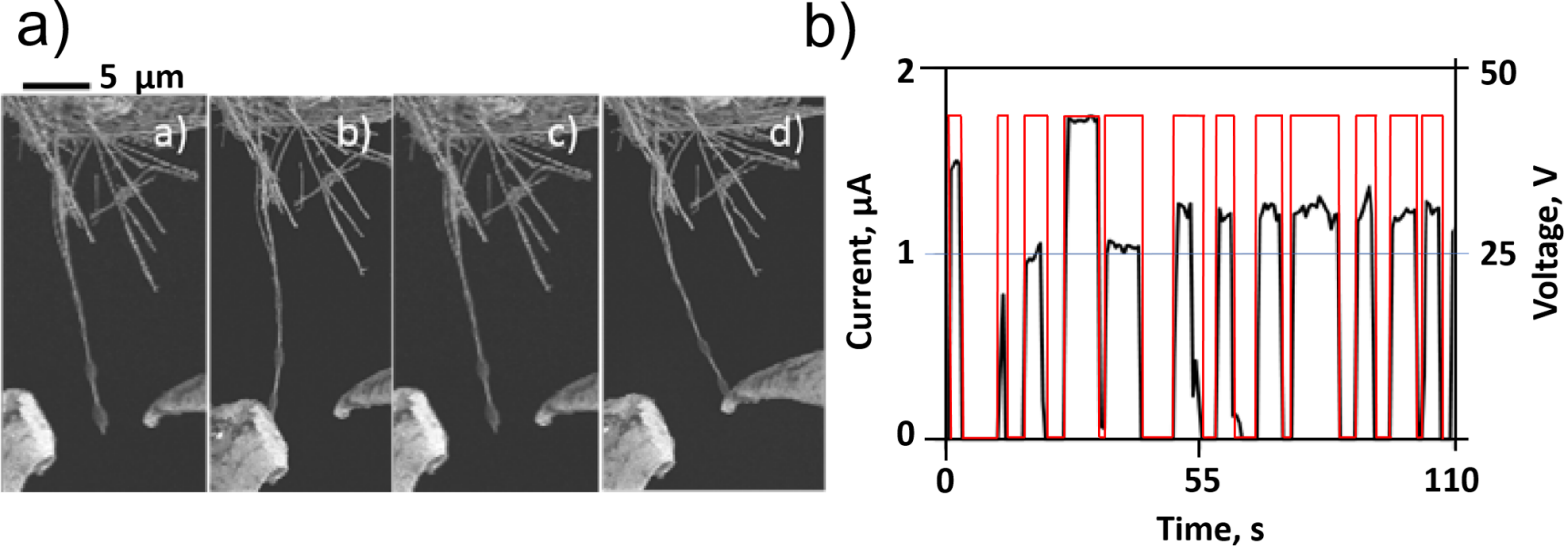
a) Example of 2T nanowire-based NEM switching between two symmetrically located drain electrodes and b) corresponding current indicating the switching events. Square voltage pulses alternately applied to the drain electrodes are represented by the red line (secondary axis). Reprinted with permission from [14], copyright 2013 Andzane et al.

A further variation of the 2T NEM switch is to use multiple drain electrodes. Recently, switching of a moving element (Ge nanowire) between two drain electrodes located symmetrically (Figure 1e,f drains 1 and 2) [14] and asymmetrically (Figure 1e,f drains 1 and 3) [11] relative to the switching element in 2T configuration was demonstrated. While switching between symmetrically located drain electrodes may be applied for switching between two circuits (Figure 4), the location of the drain electrodes at different distances from the attached end of the switching element (Figure 1e,f drains 1 and 3) allows varying of resistance, and consequently, signal strength in the circuit by adjusting the length of the switching element connected to it.

#### Three-terminal NEM switches

**Figure 5:**
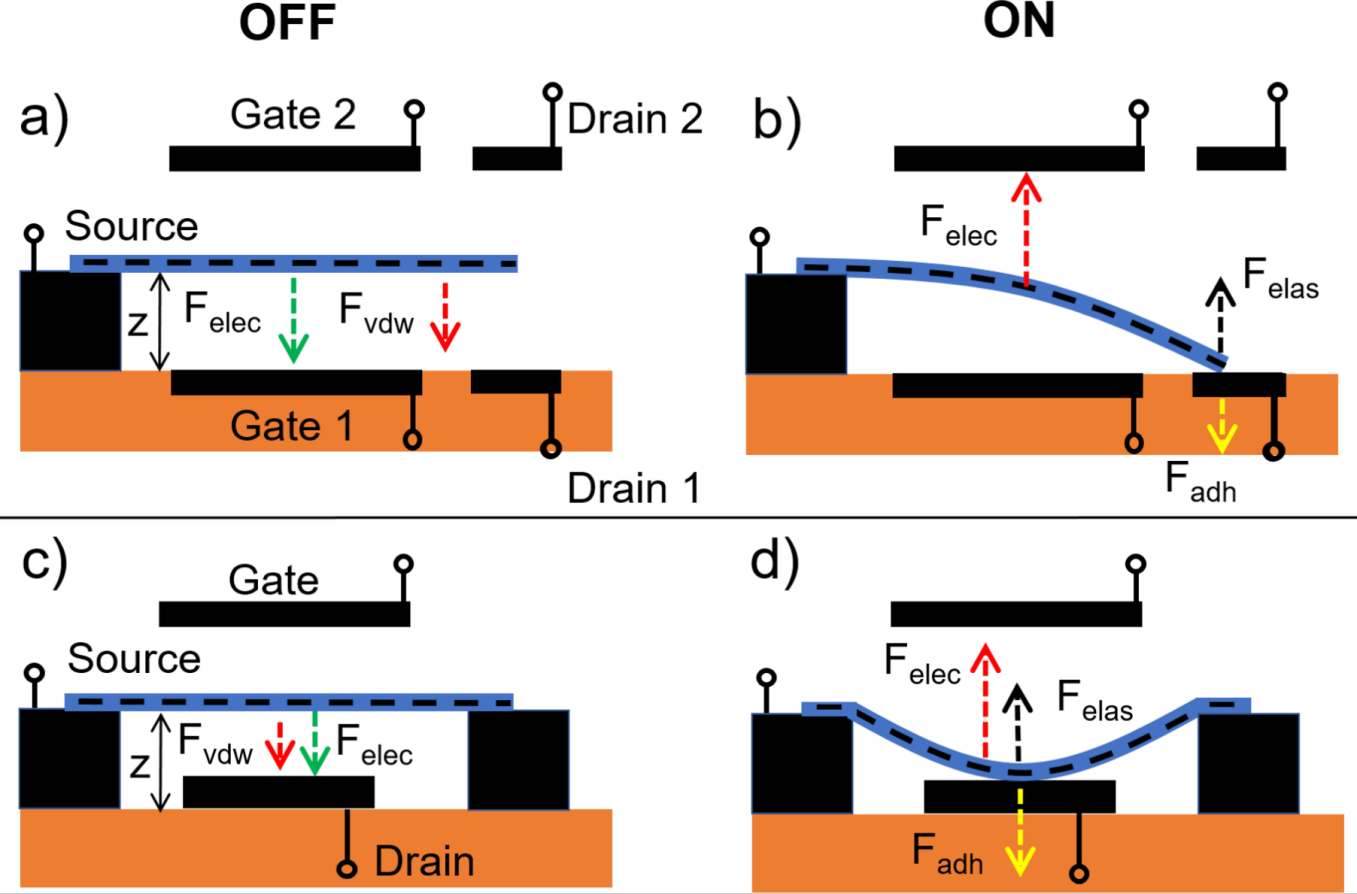
Schematics of electrostatically actuated 3T NEM nanobeam-based switches. Top panel: single-clamped 3T NEM switch in a) off and b) on state. Bottom panel: double-clamped 3T NEM switch in c) off and d) on state.

In a 3T single-clamped configuration, an additional gate electrode is used to pull the switching element in contact with the drain (Figure 5).

In comparison with 2T switches, where minimal operating voltage in the on state is fundamentally limited by the *V*_off_ voltage (Figure 2b), the use of gate electrodes allows adjustment of the source–drain voltage independent from the *V*_on_ and *V*_off_ voltages. Switching to the off position may be realized by applying the same potential to the gate 1 and drain 1, resulting in repulsive electrostatic force (Figure 5b), or by applying attractive electrostatic force between the source and the gate 2 in single-clamped configuration (Figure 5a) and between the source and the gate in double-clamped configuration (Figure 5d). In comparison with 2T NEM switches, the possibility to apply an additional restoring force to the switching element in the 3T configuration reduces the requirements to elastic properties (stiffness) of the active element necessary for switching to the off position and allows reducing the jump-in voltage by diminishing the separation gap width. However, the gap cannot be smaller than the critical distance at which vdW interactions become dominant. For nanostructures, the jump-in-contact of a switching element may occur from larger distances than theoretically predicted by the vdW interactions [53].

**Figure 6:**
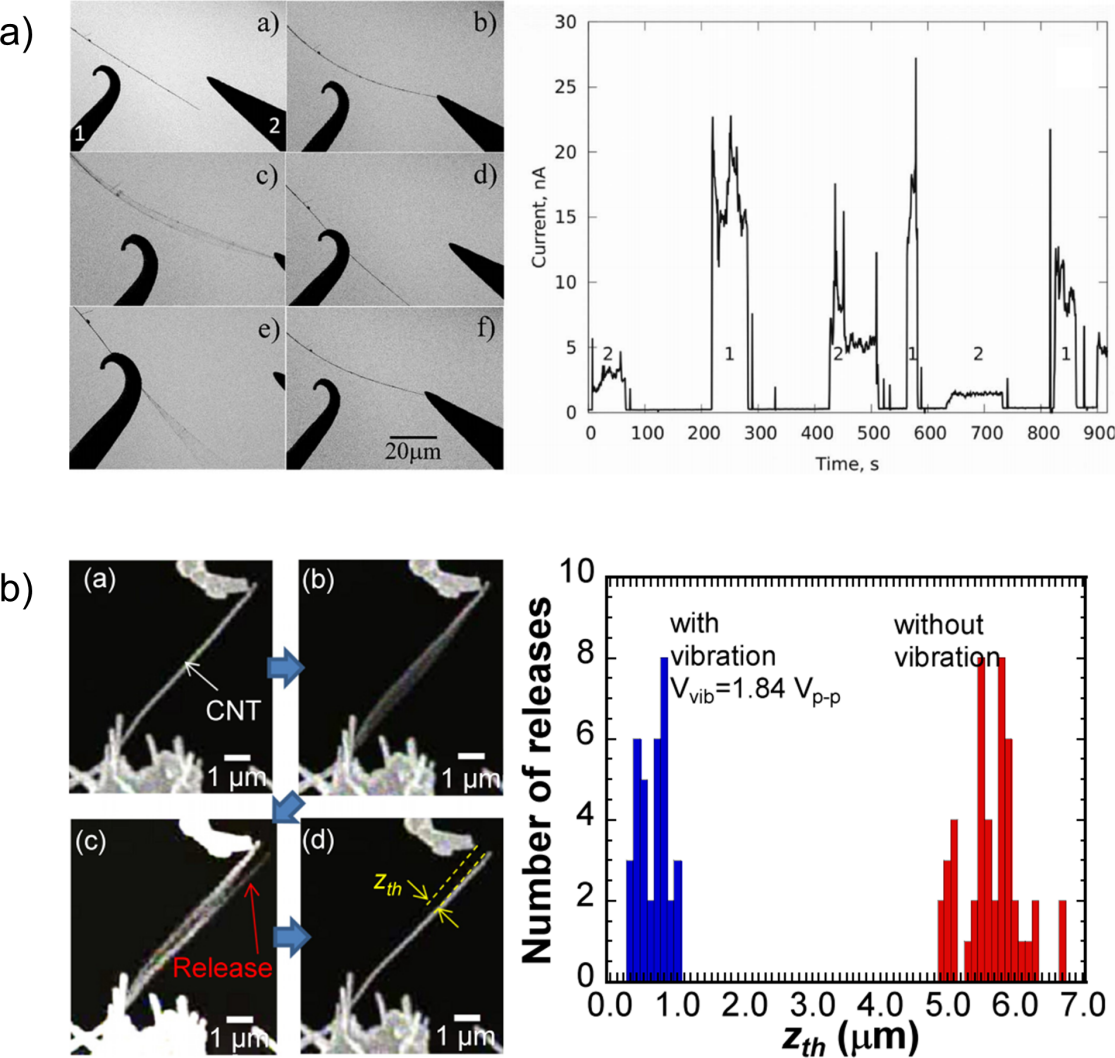
Resonant oscillations of the switching element as an effective solution to overcome on-state adhesion. a) Illustration of resonance-assisted detachment of a Ge nanowire (left panel) and corresponding electrical current measured during the nanowire switching process (right panel). Ge nanowire has been switched between two electrodes by applying a combined AC–DC field between the nanowire and the counter electrode. Reprinted with permission from [11], copyright 2013 Royal Society of Chemistry. b) Resonance-assisted release of a carbon nanotube from the contact with electrode (left panel) and corresponding reduction of separation gap (right panel). Reprinted with permission from [71], copyright 2013 AIPP.

The strong nanoscale contact adhesion, comprising an unusually strong tangential component of adhesion force [70], often poses difficulties in returning the NEM switch to its off position. Inducing resonant oscillations in the NEM switching element in the on position was found to be an effective solution for its release from the contact. The resonant oscillation modes induced in the switching element by an external AC field (Figure 6a) [11] or mechanically (Figure 6b) [71] allowed to successfully overcome the adhesion potential in the contact and allowed for reduction of the operating voltage of the NEM switch [11] or a separation gap necessary for the release of the switching element from the contact [71] by nearly an order of magnitude.

### Processes in NEM switch contacts

A reliable NEM switch contact is required to maintain stable characteristics over repetitive operation cycles. Assuming the switch is in an optimal operational environment, the contact properties are mostly defined by the contacting material properties and the real contact area. In non-vacuum environments the presence of contaminants can significantly impact these processes, as will be discussed later in this review. Despite the importance of understanding of the nanocontact evolution, only a few papers have been published with partial experimental analysis of the contact area and its influence on switching characteristics [8,10]. This section gives a brief overview of the main types of processes occurring in the nanocontacts during the operation of a NEM switch.

#### Mechanical contact

The size of the mechanical contact determines the adhesion force (*F*_adh_) value which is responsible for keeping the contacting surfaces together. Fundamentally, the contact is formed by atoms interacting across the contact interface. At the nanoscale, many models of nanocontact behaviour are based on a single-asperity model, where contacting elements are represented by single asperities with curvature radii from tens of nanometres to micrometres and are assumed to be ideally smooth [72].

According to adhesion theories [73–76], the adhesion force can be evaluated as *F*_adh_ ≈ *R*·Δγ, where *R* is radius of contact area and Δγ is energy of adhesion: Δγ *=* γ_1_ + γ_2_ − γ_12_, where γ_1_ and γ_2_ are the surface energy of the contacting surfaces, and γ_12_ is the interfacial energy in the contact. γ_12_ = 0 if both surfaces are of the same material. Thus, the surface energy of the contacting materials is a critical factor in determining the strength of adhesion in the contact in line with the contact area.

It should be noted that a real contact may consist of a number of smaller asperities (multiasperity contact) so the true contact area is smaller than predicted by the single-asperity theory. However, simulations performed by Mo et al. [77] for multiasperity contacts showed an excellent fit of this model with experimental data and the widely used Maugis–Dugdale single asperity adhesive theory model [78]. Despite the fact that such a good fit may be a result of the flexibility of the Maugis–Dugdale model, which masks its deficiencies [77], this model is a useful tool for the evaluation of adhesion in nanocontacts.

Estimations of nanocontact areas of 2T NEM switches with Ge and Mo_6_S_3_I_6_ nanowires as switching elements and Au contact electrodes [8,10], performed using a convenient approximation for the Maugis–Dugdale theory of adhesion [74] proposed by Carpick, Ogletree and Salmeron [73], showed that the typical contact area between the switching element and the Au contact electrode is 30–50 and 400–700 times smaller than the cross-sectional area of Ge and Mo_6_S_3_I_6_ switching elements, respectively [8,10] (Table 1). Thus, the contact area experiences higher current density than that inside the nanowire. This should be taken into account during analysis performed on NEM switch operation.

**Table 1:** Parameters of nanocontact areas (nanowire radius *r*_nw_, cross-sectional area of the nanowire *S*_nw_, radius of electrode apex *R*_e_, nanocontact area *S*_c_) in Ge and Mo_6_S_3_I_6_ nanowire-based 2T NEM contact switches.

Material	*r*_nw_ (nm)	*S*_nw_ (nm^2^∙10^3^)	*R*_e_ (nm)	*S*_c_, (nm^2^)	*S*_nw_/*S*_c_

Ge [10]	30	2.8	100	78	36
	150	70.7	115	331	214
	75	17.7	420	321	55
	50	7.9	1300	259	30
	60	11.3	600	218	52
Ge [11]	50	7.9	–	965	8
Mo_6_S_3_I_6_ [8]	100	31.4	–	100	314
	100	31.4	–	45	698

**Figure 7:**
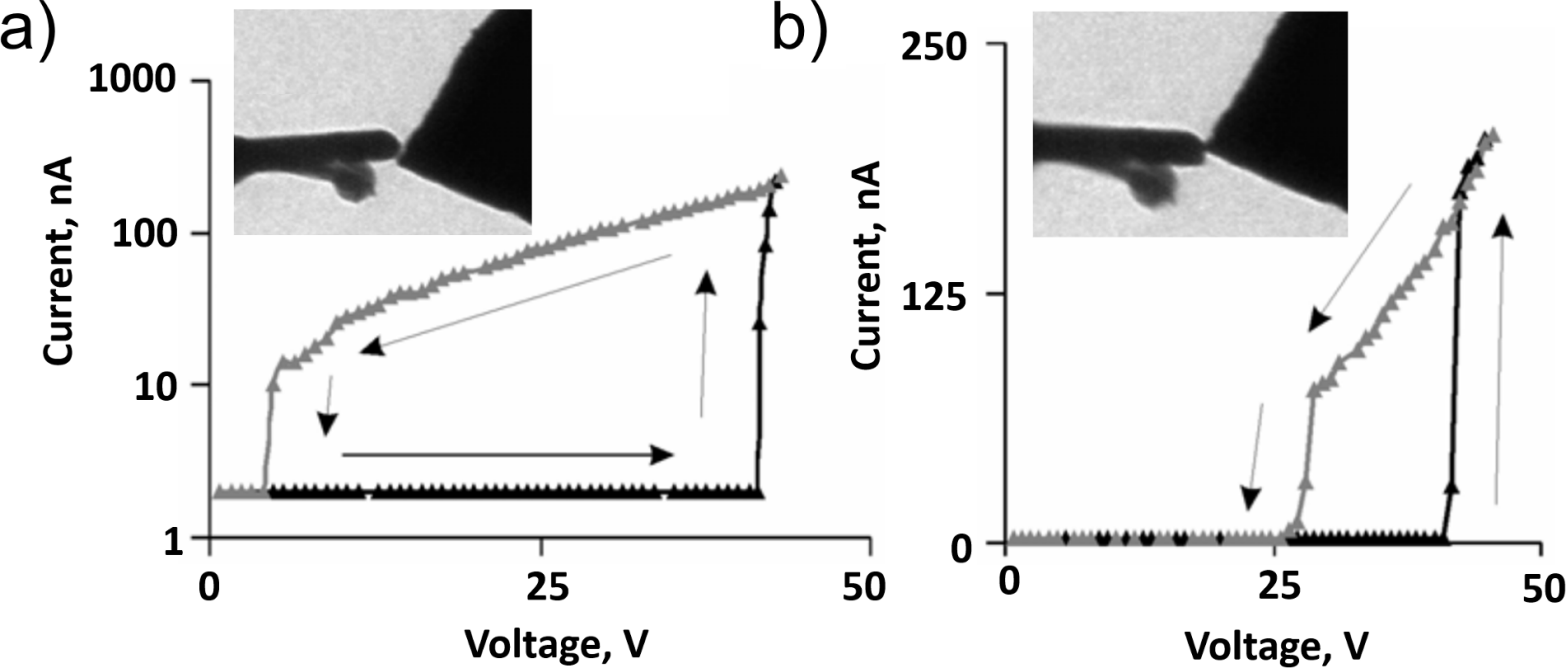
Switching cycles of Mo_6_S_3_I_6_ nanowire-based NEM switch illustrating the impact of the contact area on hysteresis width: a) *V*_on_–*V*_off_ hysteresis loop for the contact area of 100 nm^2^ (shown in the inset), and b) for the contact area of 45 nm^2^ (shown in the inset). Reprinted with permission from [8], copyright 2010 IOP Publishing.

The nanocontact area and stiffness of the switching element determine the on–off hysteresis width of a NEM switch. With the same switching element, reduction of the contact area in 2T NEM switches results in a decrease of adhesion in the contact and consequent decrease of the hysteresis width, allowing reduction of the separation gap *z* for jump-in at lower voltages. Figure 7 illustrates the decrease of the hysteresis width for a Mo_6_S_3_I_6_ nanowire bundle-based 2T NEM switch, when the Mo_6_S_3_I_6_ –Au contact area was reduced from 100 nm^2^ down to 45 nm^2^ while maintaining the same *V*_on_ voltage.

However, such reduction of the contact area results in an increase of the electrical current density flowing through it, which may lead to modification of the properties of the contacting materials. Alternatively, modifying the stiffness of the switching element can shift the jump-in and jump-off voltages and thus change the on–off hysteretic loop.

Adhesion in the contact may be impacted by the surface wear occurring during repetitive on–off switching. An AFM-based study on the nanoscale wear of diamond-like carbon against and ultra-nanocrystalline diamond showed that the surface wear increases the size of the contact by gradually removing atoms at discrete sites and is a thermally activated stress-assisted process [79]. This experiment was carried out with an AFM in amplitude modulation mode complemented with molecular dynamics simulations. An exponential wear rate dependence on the peak force load was found, suggesting that lower contact forces are needed to reduce the wear rate. It should be noted that for soft materials, plastic deformation may have a bigger effect than wear.

The presence of chemically active elements at the contact interface may also significantly increase the adhesion in nanocontacts due to formation of chemical bonds (e.g., C–Au bonds [80], Au–S bonds [8,81]**)** between the contacting materials. Nearly an order of magnitude increase in adhesion force was reported in case of covalent Au–S bond formation for Mo_6_S_3_I_6_ nanowire-based NEM 2T switches [8].

#### Electrical field and current induced processes in the contact

The electrical characteristics and performance of a NEM switch are determined by the electrical properties of the contact area between the NEM switching element and electrode. It should be noted that the true contact area does not need to be continuous, which becomes relevant when contact areas approach the size of the mean free path length of the electrons (e.g., for gold nanocontacts – 3.8 nm [82]). When the contact area becomes smaller than the mean free path of the electrons in the material, the electron transport enters ballistic conduction regime [83]. Nevertheless, typically, the metal–metal contact shows ohmic characteristics, which are preferable for low-power NEM switches, but carry the risk of switch failure at higher operation voltages.

In NEM switches with metal–semiconductor contacts, the type of the contact is determined by the mutual arrangement of the Fermi level of the metal and the valence and conduction bands of the semiconductor. The contact has a Schottky barrier if the Fermi level of the metal falls in between the valence and conduction bands of the semiconductor. The type of the contact (ohmic or Schottky) between two semiconductors is determined by the Fermi energies of contacting materials.

The presence of a nonconductive oxide layer between the contacting materials (metal–metal, metal–semiconductor, and semiconductor–semiconductor) always results in the formation of a tunnel barrier in the contact.

In the presence of a potential barrier between the contacting NEM switch materials, the magnitude of the contact resistance depends on the width and height of the barrier. In general, the interfacial charge carrier transfer in the NEM contact can be based on two different mechanisms: thermionic emission [84–85], which is dominant at high temperatures, and quantum mechanical tunnelling of carriers across the barrier width – direct [85–86] and Fowler–Nordheim (FN) tunnelling [54,85,87–88]. The direct tunnelling occurs when the barrier is trapezoidal, and the FN tunnelling occurs when the barrier is triangular [10,89–90]. The shape of the initial potential barrier in the NEM switch contact depends on the size and topography of the contact area, as well as on the band structure of contacting materials [86], and can be modulated by an applied source–drain bias in the on state of a NEM switch. For example, the change in the transport mechanism from direct tunnelling at low drain bias to FN tunnelling at the higher drain bias was shown for a Pd–MoS_2_ interface at low (123 K) temperatures [85].

**Figure 8:**
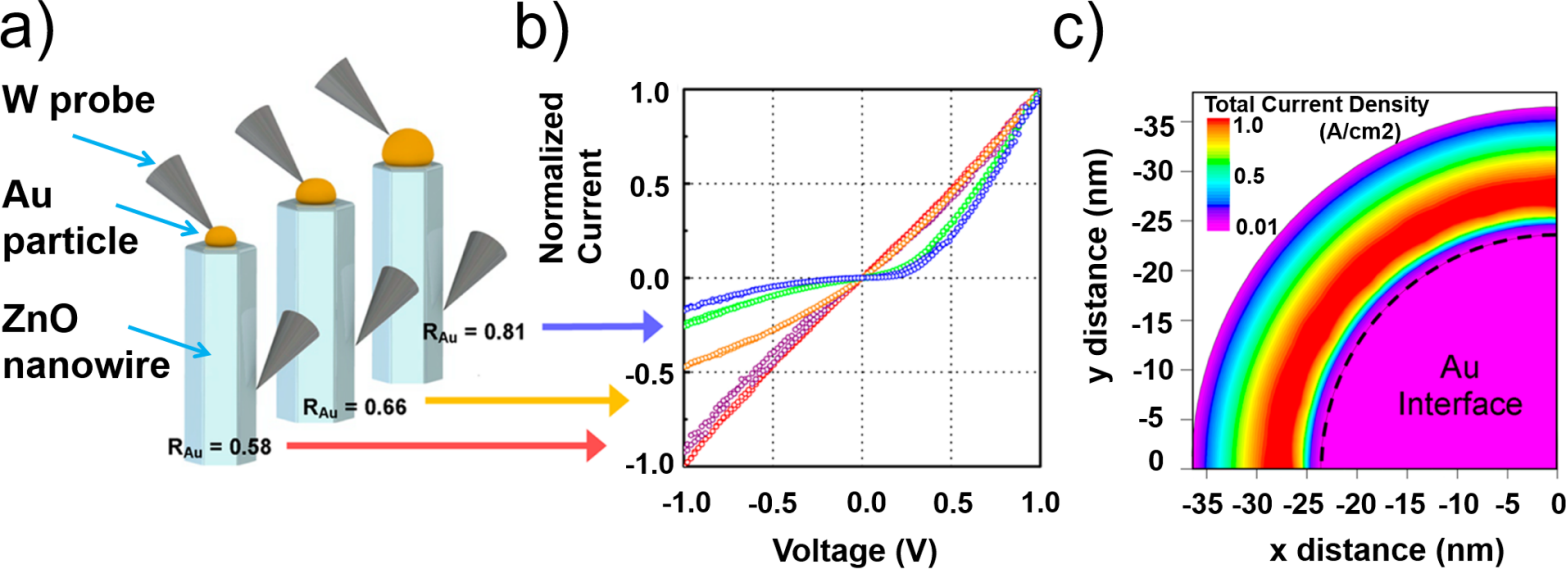
Size-dependent tuning of the mechanism of electrical conduction through a nanocontact. a) Experimental measurements of an Au–ZnO interface carried out for different Au particle/ZnO nanowire diameter ratios *R*_Au_; b) Corresponding *I*(*V*) characteristics. c) A model of the current density of the top face of a nanowire with diameter of 60 nm and *R*_Au_ = 0.8. The dashed line is the edge of the contact interface. Adapted with permission from [91], copyright 2015 American Chemical Society.

Studies of intimate ZnO nanowire–Au contacts have also shown that the mechanism of nanoscale electrical transport through the potential barrier depends on the relation between contact area and diameter of the nanowire, allowing a controllable transition from Schottky to ohmic type of conduction for smaller contact area/nanowire diameter ratios (Figure 8a,b) [91]. This effect was explained by enhanced tunnelling at the contact edge as a result of the reduction of the depletion region (Figure 8c) [91]. This allows a good ohmic contact to be obtained at a semiconductor/metal interface by only changing the contact area/nanowire diameter ratio.

As soon as the electric current begins to flow in the NEM switch contact, it causes thermal heating in the switching element and may result in small structural modifications of the contacting surfaces, welding and even change of the chemical composition of the contacting materials. Electrical-current-induced thermal effects have been studied in various one-dimensional nanostructures such as Si [92], Ge [10,54], carbon nanotubes (CNTs) [93–95], GaN [96–97] and ZnTe [98].

**Figure 9:**
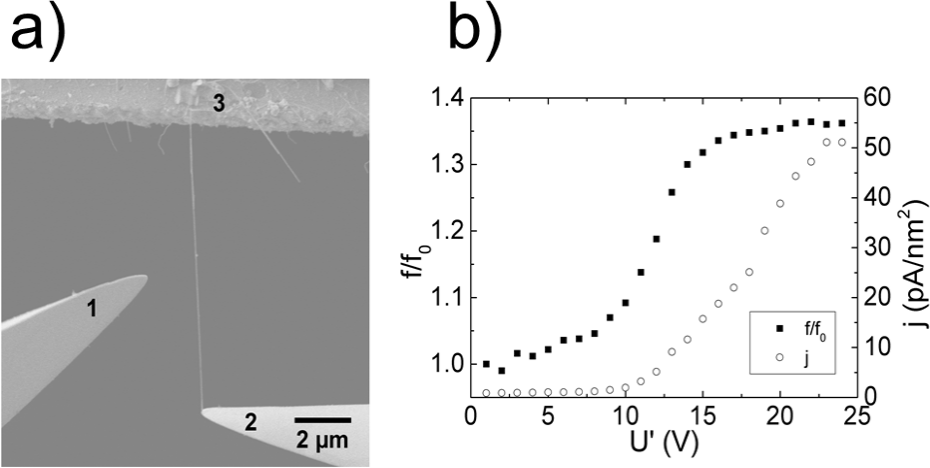
a) Experimental setup for in situ SEM investigation of processes occurring in nanocontacts. b) Graph illustrating the relative change of the resonance frequency of the nanowire and current density *j* in the contact area versus voltage applied to the nanowire. Reprinted with permission from [54], copyright 2015 IOP Publishing.

The evolution of a nanocontact between a nanowire and a contact electrode may be observed and investigated using in situ methods. Recent report showed that in situ detection of the resonant frequency of the nanowire and monitoring of its shift mirrors the electrical-current-induced strengthening of the nanocontact between the Ge nanowire and the contact electrode [54] (Figure 9a). Applying a voltage between the electrodes 2 and 3 results in a current flow through the nanowire. After each voltage application, the AC field is applied between the electrode 1 and the nanowire (electrodes 2 and 3), and the resonant frequency is determined. Electric current flow through the contact causes immediate strengthening of the nanocontact, which gradually develops until the current density of 10^−3^–10^−2^ nA/nm^2^ (Figure 9b) is reached.

Self-heating behaviour in the switching element/electrode contact is determined by a combination of its electrical and thermal conductivity [10,96,99]. If there is a low current density in the contact (in the range of 1 pA/nm^2^) and a good thermal contact, the temperature changes modestly (Δ*T* < 30 K) and almost no Joule heating occurs [10]. However, even at current densities as low as 3 pA/nm^2^, energy dissipation in the contact may result in a smoothening of the contacting surfaces because of local Joule heating and welding. The contact strengthening effect in Figure 9b was explained by smoothing and thinning of the native Ge oxide layer which results in an increase of contact area and adhesion force [54].

At higher current densities (1–10 nA/nm^2^) a Joule heating induced rise of temperature (in the range of 1000 °C) may trigger a change of chemical composition of the material. For example, for Mo_6_S_3_I_6_ bundles, a non-reversible transformation to Mo was reported, as a result of evaporation of S and I [100]. This effect may be used to anneal nanowires for enhancing electrical and field emission properties [100].

Current densities higher than 10 nA/nm^2^ were reported to be applicable for Joule heating induced local welding of Ag (12 nA/nm^2^) [101] and Pt (up to 14.5 nA/nm^2^) [102–103] nanowires, as well as for welding of dissimilar materials.

To avoid high current densities, which is mostly a concern in 2T NEM switches operating at high voltages, a thin insulating layer between the contacting surfaces has been used [10,13,50]. Another solution for the limitation of the current density at the jump-in moment for NEM switches is the connection of series resistance of 25–500 MΩ in the circuit [8,10,13,17].

The electrical field between NEM switch elements may give rise to material transfer [104–105]. During switching to the on state, a reduction of the separation gap between the electrodes prior to their mechanical contact results in an increase of the electrostatic field between them, especially at their highest asperities [105], where the electrostatic field achieves high enough (≈10^8^ V/m) values to induce FN electron emission from the electrode (cathode). This process causes a temperature increase of the other electrode (anode), resulting in thermal evaporation of the electrode material and its transfer to the opposite electrode. For metal electrodes, the material transfer issues are reported for the source–drain bias exceeding 5 V. When the contact electrodes are made of two different materials [106], material transfer results in an increase of adhesion in the contact and also makes the surfaces rougher, thus increasing resistance in the contact. For self-mated contacts, for example, in an all-molybdenum switch, material transfer was reported to be a possible cause for the observed contact resistance rise after approximately 10^4^ switching cycles operating at 1 V drain voltage in a 3T configuration with 100 nm gap between the beam and the drain electrode [19].

### Choice of material for the NEM switching element

The material properties (Young’s modulus, free surface energy, electrical conductivity, melting temperature) govern device performance (switching speed, range of operational voltage and current, reliability and durability), as well as dictate suitable fabrication approaches. The following subsections provide a brief overview of materials and material combinations used in the fabrication of active elements of NEM contact switches.

A comparison of the physical properties of the materials, together with the reported fabrication approaches, advantages and challenges for each of the material classes, and their prospective applications, summarizes the overviewed materials for NEM switching elements in a concluding subsection.

#### Metals

Metal–metal contacts in NEM switches are particularly advantageous for radio frequency (RF) applications due to their low electrical resistance. Experimental studies of elastic properties of metals, supported by atomistic simulations, have revealed several different ways the size depends on elastic properties: (1) increase in the Young’s modulus of metallic nanowires relative to the bulk value of the metal, as their diameters are reduced (e.g., Ag and Pd [107–109] nanowires); (2) decrease of Young’s modulus with decreasing size, for example, for Cr nanocantilevers [110]; (3) Young’s modulus shows almost no dependence on the diameter of metal nanowires, for example, for Au [111]. The change of the Young’s modulus can be explained by an increased influence of the surface atoms on the overall elastic behaviour of the nanostructure at sizes below a few nanometres [112–113], or by the noncrystalline structure of the studied samples [110]. Atomistic simulations for fcc noble metals [113] showed that either a decrease or increase of the Young’s modulus for metallic crystalline nanowires can be achieved by variation of their size and operation temperature. The possibility to tune the Young’s modulus by changing an element size combined with facile integration with existing complementary metal oxide semiconductor (CMOS) devices, make metals attractive candidates for the use in NEM switches. However, metal-based NEM components with nanometre-scale dimensions are difficult to fabricate due to their high intrinsic stress, large surface roughness and grain size, inherent porosity, and low strength. To date, there are rather few reports on metallic NEM switches [17–18,114–115].

Molybdenum is attractive as a NEM switch material due to its high melting temperature (2622 °C [116]) and Young’s modulus (290-380 GPa) [117]. Recent reports on Mo-based NEM switches have proven the robustness of the material. An all-molybdenum 3T NEM switch was fabricated by a top-down approach by filling Mo into a SiO_2_ mold, prepared by a one-mask photolithography process. This process was followed by etching of the SiO_2_ sacrificial layer for the release of Mo switching structures [19,115]. Switches with 300 nm thick and 500–700 nm wide switching elements with lengths 28–40 µm showed jump-in voltages in the range of 12–24 V for separation gaps of 100–150 nm. Cycling tests performed with Mo-based switches showed 100% repeatable operation of the switch with aforementioned dimensions of the switching element [115], as well as stable operation up to 20,000 switching cycles in vacuum at 300 °C [19]. The low subthreshold swing of 2.5 mV/decade was kept until the very end of the cycling tests [19]. The reliability of Mo-based NEM switches was found to be size-dependent due to the influence of residual stress in the material on the shape of the switching element.

Copper has been used as a NEM switch structural material to increase the feasibility of fabrication benefitting from a commercial CMOS technology [17]. 2T switches have been fabricated using the back end of line (BEOL) Cu layers of a commercial 65 nm CMOS technology. As-fabricated NEM switches showed jump-in voltages as low as 5.5 V, a good on/off ratio (10^3^), and high miniaturisation level, surpassing other [17,23,49,114] top-down NEM switch fabrication approaches.

Similarly to copper, the fabrication of a platinum cantilever NEM switching element involved an additional thermal annealing step at 300 °C to reduce the stress gradient in the beam. The usability of platinum for electron-beam lithography-based fabrication of NEM relay-only and CMOS–NEM hybrid circuits was demonstrated. Platinum cantilevers with thickness of ≈60–70 nm, length of ≈3.2–3.5 μm and gap of 100 nm, showed a jump-in voltage of 3.3 V in 3T configuration, and 5–6 V in the CMOS–NEM hybrid circuit [114].

Ruthenium also allows CMOS compatible fabrication in addition to such benefits as high stiffness (Young’s modulus 447 GPa), hardness (5 GPa), and high melting point (2333 °C [116]). Ru-based NEM relays with small gap widths have shown an even smaller coupling area than that of Cu-based devices, with a pull-in voltage of 5 V [18], together with the best-achieved turn-on delay of 400 ns. When tested for durability, the Ru device withstood more than 2 × 10^6^ switching cycles at 1 kHz frequency. However, when downscaling Ru NEM switches, residual stress must be accurately controlled to avoid the buckling of beams after etching of the sacrificial layer. Another concern is the edge roughness of the as-fabricated beams that leads to variation in jump-in voltages and contact resistances for a given design [18].

To our knowledge, metal alloys used as NEM switching elements have been reported in only one study using a TiW switching element and W as the contact electrode [49]. A jump-in voltage lower than 1 V and on/off current ratio higher than 10^5^ were demonstrated for this 2T NEM switch, employing an innovative “pipe-clip” architecture. However, the device showed poor reliability with marked deterioration in performance after 10–20 switching cycles. This was attributed to physical degradation of contacts, as well as formation of Ti and W oxides during the device processing.

#### Carbon allotropes

**Carbon nanotubes:** Carbon nanotubes have diameters ranging from the subnanometre range to tens of nanometres and may exhibit length-to-diameter ratios of up to 132,000,000:1 [118], which is significantly larger than for any other material, and thus could offer improved sensitivity. They also possess extraordinary mechanical strength (Young’s modulus up to 1 TPa [119–120]), high thermal (2–6 kW∙m^−1^∙K^−1^ [121]**)** and electrical (10^6^–10^7^ S/m) conductivity.

Several CNT-based relays and switches have been fabricated using the bottom-up arrangement of CNTs, including dielectrophoresis [33], controlled growth of CNTs [34,37], dispersion coating [12,35–36], nanomanipulation [15,32] techniques and electron beam lithography/metal sputtering for the fabrication of electrical contacts. CNT-based NEM switches exhibit a high on/off current ratio (10^4^–10^5^) [59] and switching speed (≈1 ns) [37] in combination with a jump-in voltage that can be as low as a few volts [35,37]. Yet, most of these devices were unique laboratory scale demonstrations. To the best of our knowledge, the highest durability (10^6^ on/off cycles, 10 ns response time) of a 2T CNT-based switch was demonstrated by Loh et al. for carbon–carbon contacts, using multiwall CNT as a switching element and diamond-like carbon (DLC) as a contact material [12]. However, the reported actuation voltages for these switches were relatively high at 20–40 V. The high current density that is caused by the high actuation voltage in CNTs during switching cycles can be lowered by the use of an insulating layer [15] or by the choice of the appropriate type of CNTs. For example, studies performed on properties and breaking parameters of different types of CNTs found that bamboo-like multiwall CNTs are the most suitable for applications in NEM contact switches due to much higher (≥25 V) burn-off failure voltages than for tube-like multiwall CNTs (4–5 V) [122].

Besides individual CNTs, films consisting of many CNTs have also been employed in the fabrication of NEM switches. Networks of CNTs are currently under ongoing research for use in 2T non-volatile memory devices, to date showing good reliability of ≈10^12^ non-volatile switching cycles with no observable wear or fatigue and read–write voltages below 5 V [123]. Here, the switching to the off state is achieved by applying a voltage pulse, presumably causing a phonon heating driven repulsion force [123].

However, practical challenges for the use of CNTs in NEM contact switches still remain, such as dependence of electrical properties of CNTs on mechanical strain [124–125], their electrical breakdown and mass loss caused by field evaporation [126].

**Graphene:** For fabricating NEM switches, monolayer [24–27] as well as few-layer [28–31] graphene materials are used. The mechanical properties are decisive for selecting the number of layers of the graphene structure [26]. Few-layer graphene is more favourable for reversible switching due to higher stiffness than single-layer graphene, but it requires larger jump-in voltages as a consequence. Graphene NEM switches are fabricated using dry or polymer-assisted transfer techniques of chemical vapour deposition (CVD)-synthesized or mechanically exfoliated graphene flakes to the desired position on the substrate, and photo- and electron-beam lithography and metal sputtering techniques for fabrication of the electrical contacts. A SiO_2_ sacrificial layer may be used for the release of graphene switching elements.

**Figure 10:**
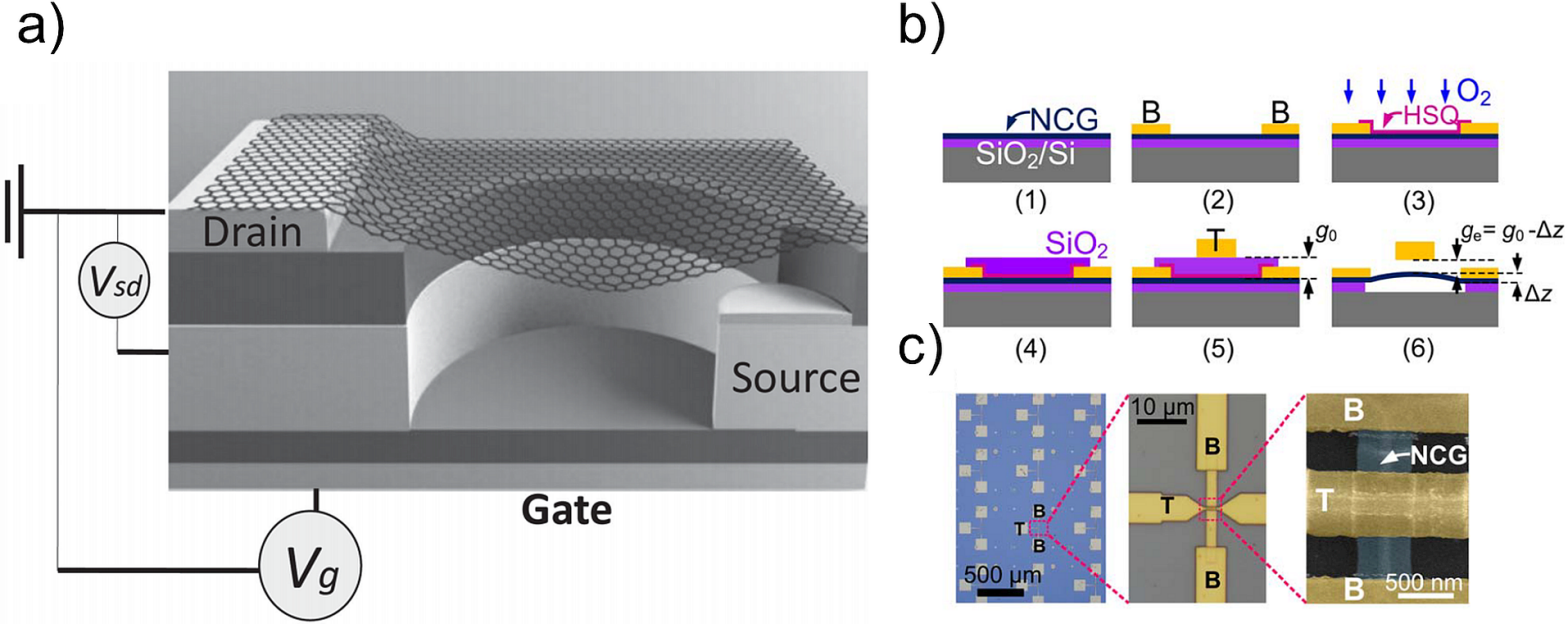
a) NEM switch with graphene sheet as the active element. Reprinted with permission from [25], copyright 2014 John Wiley & Sons, Inc. b) Fabrication process of a nanocrystalline graphene (NCG) based NEM switch with bottom (B) and top (T) electrodes. c) Optical and scanning electron microscope images of the as-fabricated device arrays. Reprinted with permission from [27], copyright 2016 Royal Society of Chemistry.

When the bending occurs along the length, and the normal stress along the width is negligible, the narrow graphene strip behaves as a nanobeam [127]. Graphene switches reported to date are primarily 2T and operate by deflecting the double-clamped graphene beam suspended over the drain electrode. Another architecture of graphene-based devices includes circularly clamped graphene switching elements [25]. Such devices can operate in 2T or 3T configuration (Figure 10a) with sub-5 V actuation voltage and provide a “line” contact of graphene membrane during switching.

The typical number of on/off cycles performed by graphene-based devices varies from 4–30 [60] up to 5000 [26]. These switches have demonstrated jump-in voltages below 1–3 V, high on/off ratios (≈10^5^) and switching speeds on the order of 100 ns. The lack of reliability of graphene-based NEM contact switches is explained as follows: currently, large area graphene can be prepared by the CVD technique; synthesized by this method, graphene has a polycrystalline nature. Processes occurring at the grain boundaries of polycrystalline graphene as, for example, charge carrier scattering and mechanical stress, result in significant degradation of graphene properties and, consequently, poor performance of the CVD-graphene-based NEM switches. Also, the Young’s modulus of CVD graphene is only about 40% of that of exfoliated pristine graphene (0.4 TPa vs 0.98 TPa [128]). CVD graphene NEM switching elements with comparable properties to mechanically exfoliated pristine graphene were fabricated from a single CVD grown graphene domain [26]. However, NEM switch fabrication using CVD synthesis of graphene is a complicated method, as it is followed by polymer-assisted graphene transfer to an insulator substrate and a microfabrication process to configure the NEM switch, posing challenges for large-scale production.

To facilitate the fabrication of graphene-based devices, direct growth of nanocrystalline graphene on insulating substrates using regular thin film process techniques (example of growth process is shown in Figure 10b) has been reported [27]. The nanocrystalline graphene on insulator had a very low thickness, good uniformity, and a Young’s modulus comparable to mechanically exfoliated graphene [27]. Both single-crystalline and nanocrystalline graphene are promising for commercial integration in high-performance NEM switches regarding their physical properties. Similar to CNT [12], graphene NEM switches can find applications for data storage and logic [28]. Currently, scalable production methods of graphene requires temperatures of ≈800 °C [27] or higher, which are still too high for CMOS integration.

#### Semiconductors

**Figure 11:**
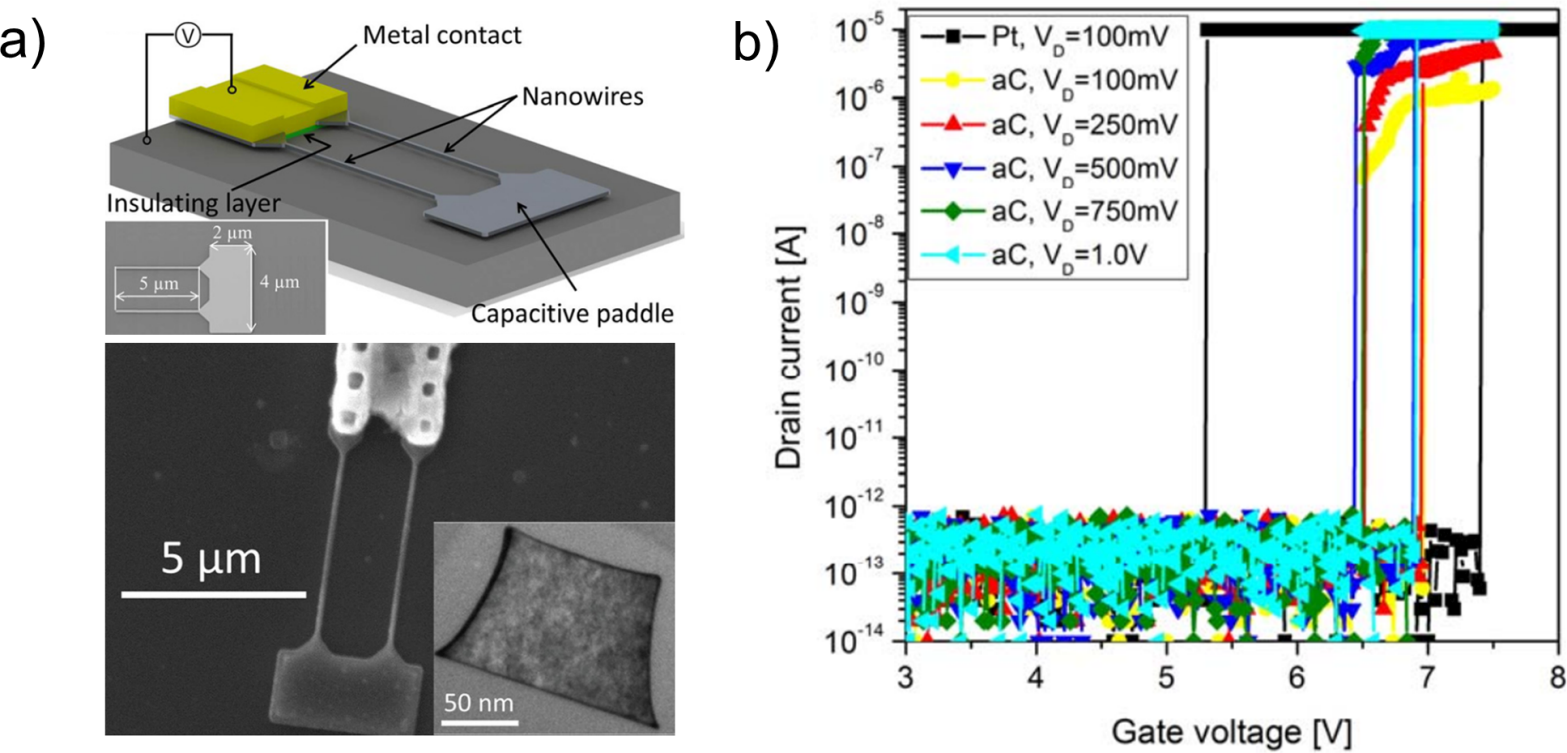
a) Schematics and design of U-shaped dual Si beam NEM switch. Reprinted with permission from [20], copyright 2012 AIPP. b) *I*(*V*) characteristics of a Si-based NEM switch illustrating significant reduction of hysteresis loop from >2 V to 0.5 V when initial Pt contact (black line) was replaced by amorphous carbon (aC) coating (coloured lines). Reprinted with permission from [61], copyright 2014 IEEE.

**Silicon and germanium:** Both Si and Ge have a long history as semiconductor device materials. Comparable relatively high Young’s moduli (Si, 130–188 GPa [129] and Ge, 103–150 GPa) [130–131] make these materials useful for applications in NEM devices. Due to the possibility of anisotropic etching, Si is widely used in top-down fabrication of NEM switches [20–22,50,132]. Top-down Si-based NEM devices of different designs (for example, U-shaped dual-beam structure with capacitive paddle (Figure 11a) [20], torsional [133]) are typically fabricated from single- or polycrystalline Si substrates using a SiO_2_ layer as a sacrificial material to release free-standing elements [20–22,50,132–133]. 3T Si-based NEM switches can operate at jump-in voltages as low as 0.8 V [20].

Both Si and Ge nanowires are used in bottom-up fabricated NEM switches and in in situ testing of devices prototypes [9–11,13–14]. It was found that the presence of a native oxide layer on Si and Ge nanostructures implies some limitation on NEM switch operation at low voltages due to the high contact resistance expressed in low on/off ratio [9] and non-conductive gap below 2–4 V [10,13,54]. On the other hand, in 2T NEM switches, native oxide covered switching elements are able to operate without breakdown at voltages up to 40 V [10], required for repeatable on/off switching.

In terms of reliability, individual Ge nanowire-based 2T devices showed good durability with no signs of degradation during tens of switching cycles [10–11]. The operation at low on-state voltages is typically achieved by coating of Si or Ge NEM elements with some other conductive material like Au or Au alloys, Pt or amorphous carbon (aC) [9,61]. For example, coating Si NWs with metal (Au/Al) resulted in an improvement of the on/off current ratio by an order of magnitude [9], but coating of a Si-based NEM switch with amorphous carbon (aC) (Figure 11b) [61] allowed more than 10^8^ switching cycles to be achieved. An alternative method of lowering the on-state voltage range is removing the native oxide layer from the nanowires’ surface [10,13].

**Molybdenum–sulfur–iodine:** Excellent functional properties of nanowires based on transition metal chalcogenide-halides, combined with easy synthesis and intrinsic absence of impurities make them attractive NEM switch elements. The properties of these materials include high electrical conductivity [134], good thermal and mechanical stability and ability to withstand high (up to 50 V) operational voltages [8]. Molybdenum–sulfur–iodine (Mo_6_S_3_I_6_) molecular wire bundles were investigated as NEM switching elements and as a contact material employing combined in situ TEM-nanomanipulation techniques aimed to determine how changes in the contact electrode material and geometry of 2T NEM switch influence the device characteristics [8]. The very low coefficient of friction of Mo_6_S_3_I_6_ (0.03 [135]) could help to reduce surface wear originating from the tangential forces in the NEM contact when switching to the on state [70].

**Silicon carbide:** Silicon carbide (SiC), well-known for its resistance to corrosion, has been widely explored for harsh environment applications where traditional semiconductor materials fail. In addition, it has tribological characteristics superior to those of Si. SiC is a wide bandgap (2.4–3.3 eV) semiconductor with a bulk Young’s modulus of 400–500 GPa [136] and high thermal conductivity on the order of 330 W∙m^−1^∙K^−1^ for bulk 3C–SiC [137], a larger than 1 MV cm^−1^ breakdown electric field as well as a high melting temperature. Regarding its elastic properties, despite the relatively large discrepancy in the results, the correlation between the diameter of SiC 1D nanostructures (down to 18 nm) and their Young’s modulus was not found. The Young’s moduli of 18–140 nm diameter SiC nanowires were determined to be in the range of 275–750 GPa [138–139], showing average value comparable with bulk values of 400–500 GPa [136]. Reported Q factors of electrically induced mechanical resonance of SiC nanowires varied from 3,500 to 160,000 [138].

**Figure 12:**
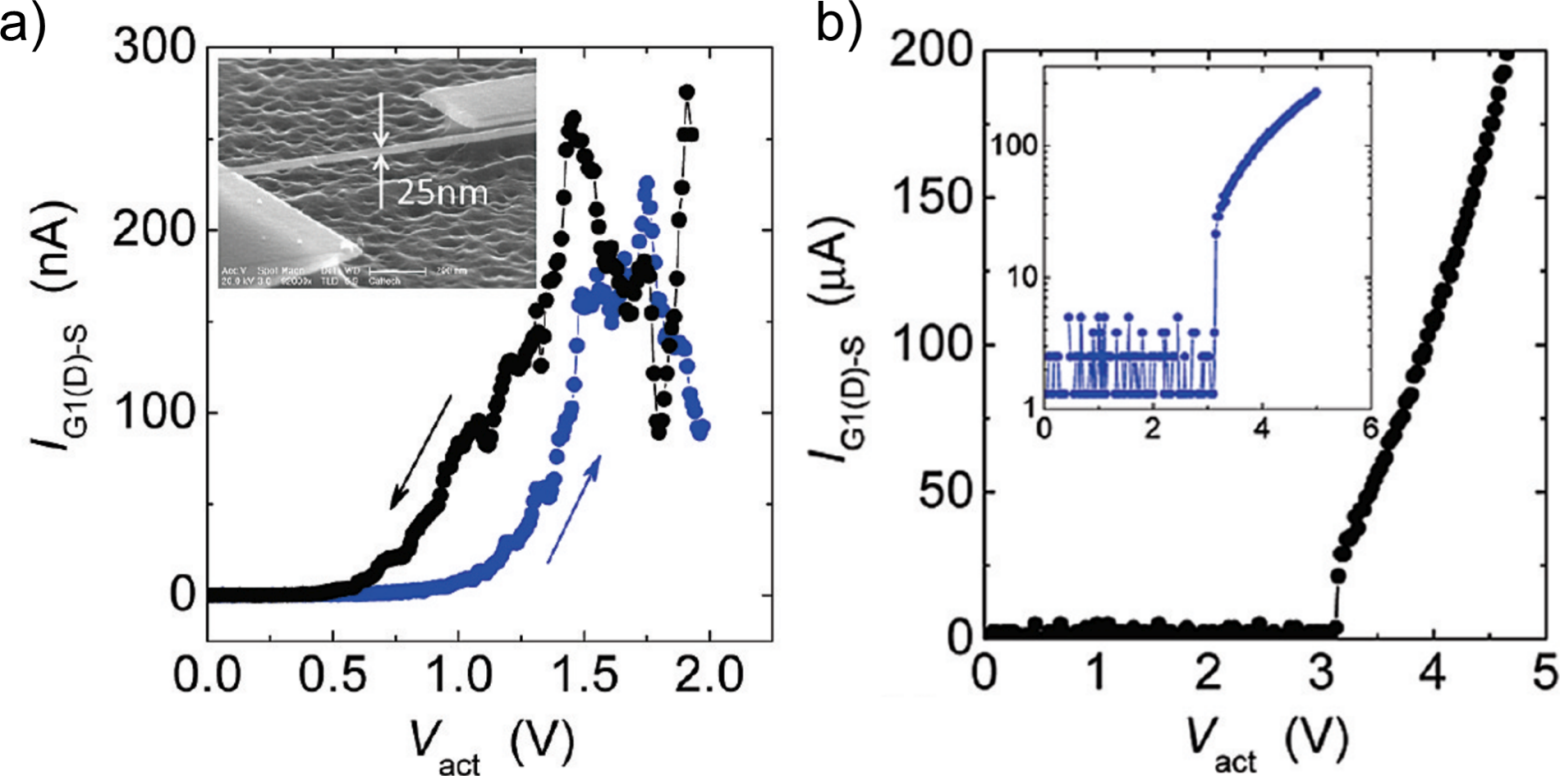
Different jump-in *I*(*V*) characteristics of a) bare, thin SiC beams (SiC–SiC contact) showing gradual current rise and b) aluminium metallized SiC (Al–Al contact) showing abrupt transition to the on state (at 3 V). Reprinted with permission from [23], copyright 2010 American Chemical Society.

SiC-based NEM switches fabricated by top-down approach have shown good durability. The approach involved electron beam lithography patterning and etching of the SiC layer deposited over the Si/SiO_2_ substrate and following release of SiC nanostructures by removing the SiO_2_ sacrificial layer. The as-fabricated 400–500 nm thick poly-SiC beam-based 3T switches showed stable operation during more than 10^6^ cycles at both room temperature and as high as 500 °C [38–39]. Reducing the size of the switching element down to 25–50 nm resulted in the gradual structural deformation of the switching element [23]. SiC NEM switches with 25–50 nm thick switching elements exhibited unstable switching to the on state, lacking a distinct current rise (Figure 12a), presumably caused by the lower conductivity of SiC–SiC contacts in comparison to metals. The metallization of SiC switches with aluminium resulted in a sharper on-state current rise (Figure 12b), but reduced durability of these NEM switches down to a few or in some cases singular switching events [23].

#### Ceramics

**Titanium and tungsten nitride ceramics:** Titanium nitride (TiN) has a low electrical resistivity that is comparable to some metals, high stiffness (Young’s modulus of 427–590 GPa) and high hardness, high melting temperature (2930 °C), high corrosion resistance [140], and low surface energy [141] – all beneficial properties for NEM switch applications. TiN is also one of the materials used in almost all standard surface and bulk micromachining, thus available from routine CMOS production, and it can be integrated in existing devices. Meanwhile TiN exhibits strong residual stress within the thin film layers which has to be taken into account for NEM switch applications as it can cause unwanted out-of-plane deformations of switching elements [6,142]. Therefore, specific treatments for switching elements such as high-temperature annealing is necessary to release the residual stress [142]. Typical actuation voltages reported for TiN NEM switches were in the range of 5 V [48] to 14 V [47]. The 35 nm thick TiN beam-based NEM switch with a TiN/W contact electrode coated with a 8 nm thin SiO_2_ layer and a 15 nm separation gap was operated in ambient air for more than 400 cycles with stable actuation voltages [47]. The jump-in voltage of TiN NEM switches can be lowered by using a harder material with higher melting temperature (for example, Al_2_O_3_) as the electrode coating [48]. Configuration of 2T TiN switches with three operation states (one off state and two on states with different jump-in voltages), presented in [48] can serve for memory and logic applications.

Amorphous tungsten nitride (WN*_x_*) was proposed by Mayet et al. [5] as a prospective high-quality structural material for top-down fabrication of NEM switches. The amorphous WN*_x_* thin film deposited using a tungsten target at room temperature has a Young’s modulus as high as 300 GPa, which is comparable to the widely used WN*_x_* protective coatings (300–390 GPa) [143]. 3T NEM switches were fabricated by a top-down approach using reactive ion sputtering over a SiO_2_ sacrificial layer, employing electron beam lithography and etching methods. Experimental results on these NEM switches, where both the switching element and the contact electrode were fabricated from WN*_x_*, indicated that this material is suitable for low-voltage switches. A NEM switch with design of relatively large dimensions (switching element size of 190 nm × 500 nm × 20 µm) [5] achieved a jump-in voltage of 0.8 V. High-contact resistance (10 MΩ) observed in the experiments indicated that these materials may be used in applications where current must be limited.

#### Comparison of NEM switching element materials

**Figure 13:**
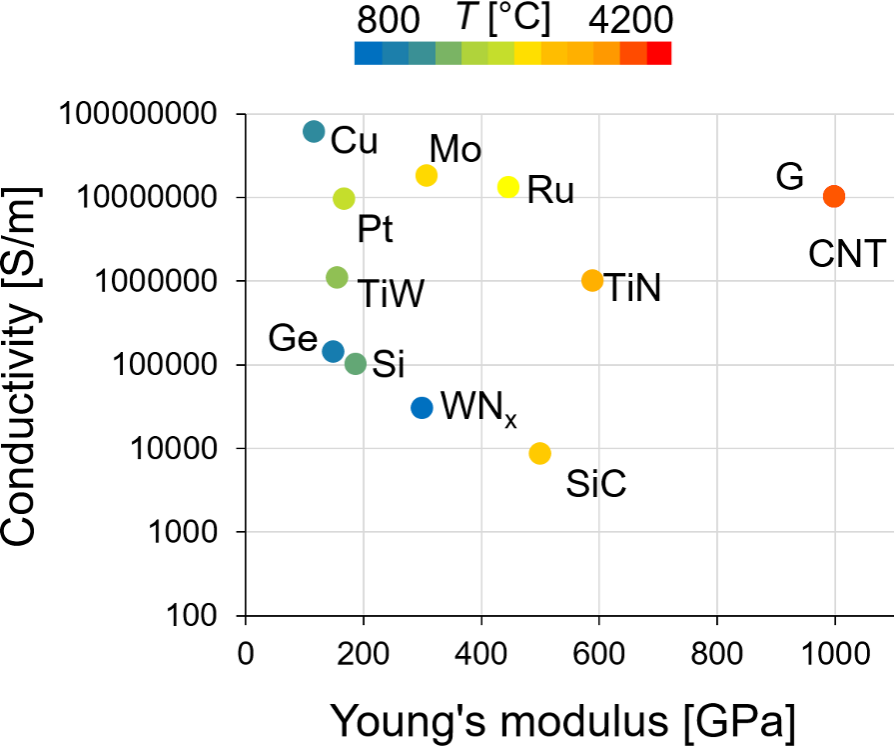
Materials properties for NEM switching elements. Marker coordinates correspond to the electrical conductivity and Young’s modulus of each material, and the marker colour represents its melting temperature *T*.

Although the above overviewed materials belong to different classes, their mechanical, electrical and thermal properties vary in a relatively narrow range (Figure 13). Typically, they can withstand high temperatures of at least 800 °C and are characterized by a Young’s modulus of hundreds of GPa and have an electrical conductivity above 10^4^ S/m. High Young’s modulus values result in switching speed and a stiffness needed for the fast and volatile operation of a NEM switch. The electrical conductivity of the switching element is decisive for the desired application. For example, for RF applications, metallic conductivity is needed, while materials with relatively low conductivity are suitable for electrical current limitation applications. The ability of withstanding high temperatures is favourable for NEM switches operating in harsh environment. A demonstrative comparison of fabrication approaches, general advantages and challenges, together with some of the proposed applications of the materials for NEM switching elements is presented in Table 2.

**Table 2:** Comparison of materials for NEM switching elements.

Materials		Device fabrication approaches (examples)	Advantages	Challenges	Possible applications

metals	Mo [19,115] Cu [17] Pt [114] Ru [18] TiW [49]	top-down (lithography, etching, thermal annealing, atomic layer deposition)	- high electrical conductivity	- surface oxidation (Mo, Cu, Ru, TiW) - scaling and grain boundary effects on mechanical properties - residual stress (Pt)	- RF applications
carbon allotropes	CNTs [12,15,32–37,59,123] graphene [24–31]	top-down (lithography, etching, metal sputtering) with bottom-up (dielectrophoresis, controlled growth, nanomanipulation)	- combination of high electrical conductivity and extraordinary mechanical strength - very high decomposition temperatures	- mechanical strain-dependent electrical properties (CNTs) - mass loss and electrical breakdown (CNTs) - grain boundary effects on mechanical and electrical properties (graphene)	- memory and logic applications
semiconductors	Si [20–22,50,132–133] Ge [9–11,13–14] Mo_6_S_3_I_6_ [8] SiC [23,38–39]	top-down (lithography, etching) or bottom-up (nanomanipulation)	- operation at relatively high voltages up to 40–50 V (Si, Ge, Mo_6_S_3_I_6_) - can withstand high temperatures (SiC)	- surface oxidation (Si, Ge) - mechanical and electrical properties may change with increasing temperature (Ge, Si)	- memory (Si) - logic and high-temperature applications (SiC)
ceramics	TiN [6,47–48,142] WN*_x_* [5]	top-down (lithography, etching, sputtering, thermal anneal, atomic layer deposition)	- combination of high electrical conductivity and stiffness (TiN) -combination of high elasticity and hardness (WN*_x_*)	residual stress (TiN)	- memory and logic applications (TiN) - electrical current limitation and low-voltage switches (WN*_x_*)

### Choice of electrical contact material

Despite numerous studies [51–52,82] on the properties of the electrical contacts at the nanoscale, there is a limited number of reports on the suitability of different materials combinations for NEM switch applications. At the same time, choosing the appropriate contact materials is one of the most important issues in scaling the NEM switch, as traditional contact materials (e.g., Pt, Au, Cu) struggle to show reliable and stable performance due to pronounced nanoscale adhesion, wear and dynamic evolution of the contact. The importance of choosing the appropriate materials is highlighted by NEM switch research, where classical switch architectures in combination with unconventional materials combinations, for example, SiC [7] and diamond-like carbon (DLC) [12] as electrical contact materials, have led to increased reliability. The following subsections summarize the advantages and drawbacks of some of the materials being used as electrical contacts in NEM switches.

#### Metals

The reliability of various metals as electrode materials has been examined both theoretically [144] and experimentally [63,145–152] using mechanical [145–146], electrical [145–146] or coupled electromechanical [63,147–152] testing experiments. The materials properties such as hardness, wear resistance, melting point, conductivity and oxidation characteristics should be considered for each particular application.

Theoretical studies have modelled the impact of NEM switch scaling on the properties of metal electrode materials, showing that a decrease in device dimensions will necessarily result in a large increase of contact resistance as well as the risk of stiction-induced failure [144]. It was found that no metal considered in this study (Ag, Al, Cu, Pt, Rh, Ru, Ti and W) can fulfil the requirements of an ideal contact simultaneously [144].

Au is a material with an exceptional conductivity and oxidation resistance. However, the reliability of pure Au–Au contacts is very low as Au is a soft material, which is likely to undergo material transfer and wear [144,153]. Alloying with another metal (for example, Ni) is a method to strengthen soft metals to increase wear resistance and reduce surface adhesion. Two-phase Au–Ni alloys (at 20% Ni) showed reduced wear rate and a small increase of contact resistance in comparison with pure Au electrodes in a MEM switch setup [150]. It was also shown that Au, alloyed with noble metals such as Pt, Rh and Ru, reduced the contamination build-up rate [147].

Other noble metals such as Ir and Pt are attractive due to their high Young’s modulus and oxidation resistance in ambient environment. Under *I*(*V*) cycling with a Cr-coated AFM tip, both materials exhibited relatively low initial contact resistances in comparison with Ni and Cr [145]. Adhesion forces were measured to be more than four times higher for the Ir/Si contact than for Pt/Si [145]. The electromechanical cycling of Pt/Pt contacts for 10^9^ cycles in ambient air showed more than three orders of magnitude increase of contact resistance accompanied by only a moderate 12% increase of adhesion force and no signs of wear-related degradation [63]. TEM observations revealed an added layer of material on the switching element and molecular dynamics simulations in the same study showed that the presence of a tribopolymer layer between metal contacts would increase the contact resistance, but weaken the adhesive interactions, in comparison with a case with no tribopolymer.

The use of W as the contact material in NEM switches is advantageous due to the high hardness of the metal, high Young’s modulus and high melting and boiling points. A W/Cr contact demonstrated the lowest adhesion force, compared to other metals (e.g., Pt and Ni) [145]. However, for a W contact material, relatively large changes in contact resistance [146] and degradation of the on state current during cycling [49] have been reported, which has been attributed to the impact of its native oxide [49,146].

Al and Cu electrode materials have demonstrated very low initial contact resistances during *I*(*V*) cycling [145] due to the high conductivity of these electrode materials. However, both materials displayed either gradual (Al) or abrupt (Cu) increase of contact resistance after 10^4^ *I*(*V*) cycles [145].

Ti stands out with the combination of a hardness and Young's modulus relatively better than for other materials (e.g., Au, Al, Cu), showing reliable *I*(*V*) cycling with low initial contact resistance [145]. However, the stable cycling performance is compromised by its larger resistivity in comparison with other metals.

Metals have been alloyed with semiconductors in search for novel NEM switch contact materials. Platinum silicide (Pt*_x_*Si) thin films were proposed as the perspective contact material due to the combination of mechanical robustness and metal-like conductivity [63,154–155].

In summary, the highest reliability is achieved using a material with a combination of high Young’s modulus, high hardness and high melting point, for example, Ti and W, while materials traditionally used for contacts (noble metals such as Au and Pt) are at risk of wear due to their low hardness and low Young’s modulus [153].

#### Other materials

Electrically conductive stable oxides could also be considered as prospective NEM switch contact materials, as they have shown reliable performance for microscale contacts. For example, switches based on ruthenium oxide RuO_2_–Au contacts [149,151–152] reached more than 10^10^ switching cycles without failure, surpassing such materials combinations as Pt–Au and Ir–Au [149]. As RuO_2_ has lower surface reactivity than, for example, Pt, the RuO_2_–Au contact material combination prevents cycling-induced tribopolymer accumulation [149]. However, the conductivity of RuO_2_ is lower than that of Au [152].

Another perspective contact material is Mo_6_S_3_I_6_, because of its low surface energy [134]. Mo_6_S_3_I_6_ was tested in a NEM switch, where the switching element was Mo_6_S_3_I_6_ nanowire bundles, and the contact electrode material was Mo_6_S_3_I_6_ or Au. The performed tests showed reduction of adhesion per unit area by nine times for a Mo_6_S_3_I_6_–Mo_6_S_3_I_6_ contact in comparison with a Mo_6_S_3_I_6_–Au contact [8].

Diamond-like carbon (DLC) and SiC have been suggested as electrode materials due to their mechanical robustness, demonstrating stable contact resistance over 10^6^ switching cycles for CNT-based NEM switch [12] and over 10^7^ cycles for SiC-based NEM switch [7]. The applications of these materials are, however, limited due to their high contact resistance.

Table 3 presents the advantages and drawbacks of the contact materials combinations discussed above, as well as their cycling behaviour, together with experimental setups used for testing.

**Table 3:** Advantages and disadvantages of contact material/switching element combinations.

Contact material/switching element	Advantages	Cycling performance	Drawbacks

Ir/Cr [145]^a^; Ir/Pt [146]^a^	- high hardness - high resistance to native oxide formation	good cycling characteristics for 10^5^–10^9^ [63,145] cycles; low initial contact	high adhesion force^a^ in comparison with Cr, Ni, Ti, W, Pt [145]
Pt/Cr [145]; Pt/Pt [63]^a^, [144,146]	- lower adhesion force in comparison with Ir, Ti, Ni, Cr, Al, Cu [145] - harder than Au - high resistance to native oxide formation	resistance (in comparison with Ni and Cr) [145]	contact resistance increase over time [63,145] (more than 3 orders of magnitude after 2·10^9^ switching cycles [63])
W/Cr [145]; W/TiW [49]^b^, [144]; W/Pt [146]	- lower adhesion force in comparison with Pt [145] - high hardness, high melting and boiling points (compared to, e.g., Pt)	good *I*(*V*) cycling characteristics for 10^5^ cycles [145]; larger changes in contact resistance (compared to, e.g., Ir, Ni, Pt) [146]; degradation of on state current [49]	formation of native oxide
Ti/Cr [144–145]	- combination of reliability, useful lifetime, hardness and Young's modulus relatively better than for other materials (e.g., Au, Al, Cu) - high corrosion resistance	good *I*(*V*) cycling characteristics and stable contact resistance for 10^5^ cycles [145]	higher resistivity in comparison with other metals
Ni/Cr [145]; Ni/Pt [146]	- good corrosion/oxidation resistance	poor *I*(*V*) cycling performance [145]; less than 1% change in contact resistance over 10^5^ cycles [146]	high initial contact resistance (3–5 orders of magnitude higher than W, Pt, Ti, Ir) [145]
Cr/Cr [145]	- high corrosion resistance and hardness	poor *I*(*V*) cycling performance [145]	formation of native oxide
Al/Cr [144–145]	- low initial contact resistance	gradually became nonconductive after 10^4^ *I*(*V*) cycles [145]	high adhesion force, formation of native oxide
Cu/Cr [144–145]	- low initial contact resistance	abrupt large rise in contact resistance after 10^4^ *I*(*V*) cycles [145]	high adhesion force, formation of native oxide
Au/Au [147–148,150,153]^c^	- no oxidation - very low initial electrical contact resistance	material transfer during cycling	high adhesion force, low hardness; rapid surface wear
Au–Ni alloy, 20 atom % Ni/Au [150]	- reduced wear rate in comparison with pure Au	larger number of switching cycles with stable contact resistance compared with pure Au	contact resistance higher than that of pure Au
Mo_6_S_3_I_6_/Au Mo_6_S_3_I_6_/Mo_6_S_3_I_6_ [8]^b^	- low surface energy [134]	larger number of switching cycles in comparison with Au electrode	S forms covalent bond with Au
DLC/CNT [12]^b^	- low adhesion, mechanical robustness - high electrical resistivity, high corrosion resistance	stable contact resistance over 10^6^ switching cycles	high contact resistance
SiC/SiC [7]^b^	- stable performance over testing period of 7 days in ambient air - suitability for high temperature (500 °C) measurements	stable performance over 10^7^ full switching cycles at room temperature	high contact resistance
RuO_2_/Au [149,151–152]^c^	- electrically conductive and stable oxide - extended lifetime (in comparison with Pt–Au and Ir–Au) due to catalytic behaviour	reached more than 10^9^ switching cycles without failure [149]	lower conductivity that Au–Au contacts [152]
Pt*_x_*Si [63,154–155]	- combination of mechanical robustness with metal-like conductivity - good oxidation resistance	no cycling tests performed	no cycling tests performed

^a^Metal-coated AFM tip/thin film-based nanoscale test platform. Si AFM tip was used in adhesion force measurements. ^b^Representative NEM switching device. ^c^Microscale test platform.

### Operating environment

Environmental conditions (e.g., pressure, temperature, humidity, presence of chemically active gases (among which the impact of oxygen is the most widespread and studied), contamination with carbonaceous compounds [63,156–158]) can impact both the switching element and contact materials, influencing NEM switch operational parameters such as switching speed, jump-in voltage, hysteresis width, contact resistance and adhesion. The environmental effects on the switching element may result in a delay of response or change in jump-in/jump-out voltage of the device. The most severe environmental impact is experienced in the contact region, where the dynamic relationship between contacting materials can introduce variability in electrical and mechanical NEM switch operational parameters over time.

#### Environmental damping of NEM switching element

**Figure 14:**
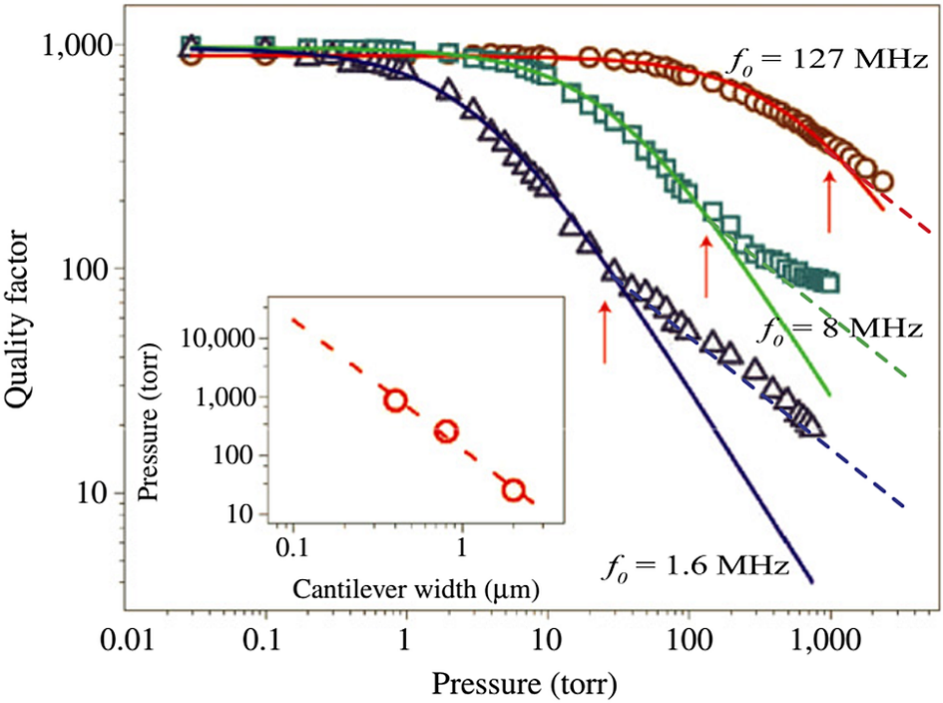
Quality factor as a function of air pressure showing the transition from molecular (solid lines) to viscous (dashed lines) damping. Red, green and blue colours mark three different sized nanocantilevers (corresponding to widths 400 nm, 800 nm and 2 µm). Inset shows the crossover pressure dependence on cantilever width. Reprinted with permission from [159], copyright 2007 Macmillan Publishers Limited.

In contrary to high-vacuum conditions, where the damping of the switching element is determined by intrinsic losses (e.g., imperfect clamping, structural defects in the switching element), in higher pressure (low vacuum, dry gas or liquid) environments it experiences additional external losses, depending on the pressure and viscosity of the surrounding medium, which are mirrored by a decrease of quality factor of the switching element and may lead to reduction of the switching speed. Figure 14 illustrates the impact of air pressure on the quality factor for three different sized SiC nanocantilevers indicating the transition from molecular to viscous damping [159], governed by collisions and viscosity of the medium, respectively.

Despite the negative impact on the quality factor of the switching element, operating a NEM switch in a water-free dielectric liquid with large dielectric constant has several significant advantages such as reduction of pull-in voltage, suppressed arcing, avoiding capillary forces and exposure of NEM switch components to oxygen, as well as reduction of van der Waals force in the contact [160]. Operation in insulating liquid media has been demonstrated for a few NEM/MEM switches. A top-down fabricated 3T NEM switch using a moving TiN cantilever and TiN electrode with a 40 nm gap operating in insulating transformer oil showed about 40% decrease in pull-in voltage due to the liquid’s large dielectric constant [160]. The additional benefits were reduction in hysteresis width and improved cycling characteristics when compared to operation in air [160]. For a much larger microelectromechanical switch, a similar 31.8% reduction in pull-in voltage and a more than 94% decrease of the adhesion force have been reported when operating in mineral oil [161].

#### Operation in humid environments and ambient air

**Figure 15:**
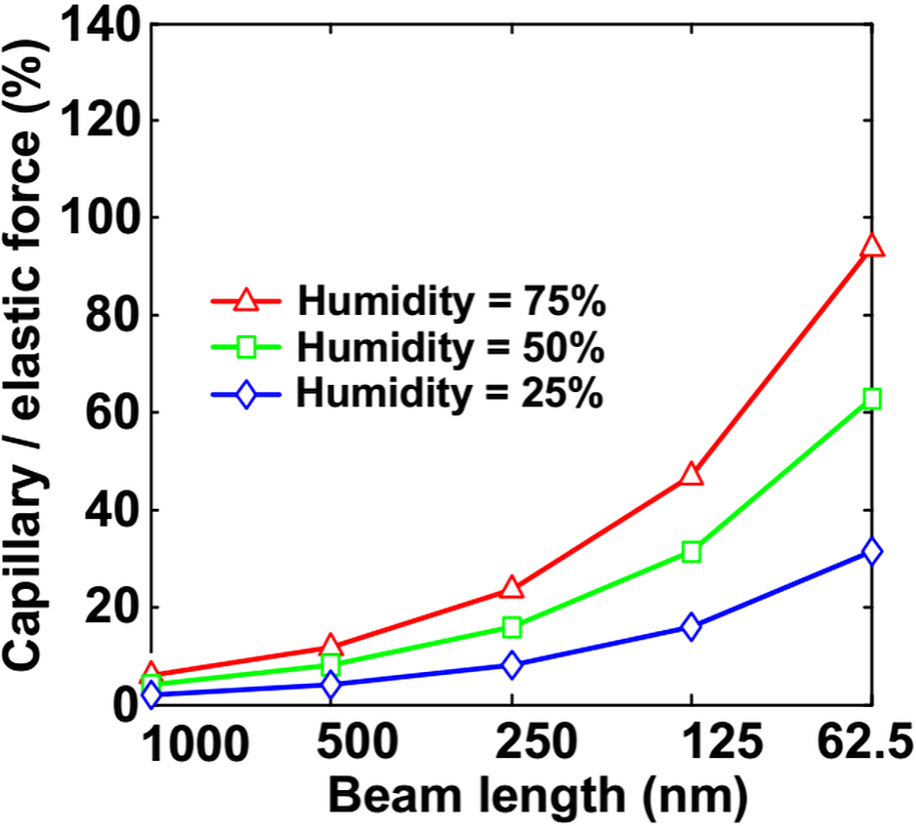
Scaling impact on the capillary/elastic force ratio. The impact of reducing the length of the switching element on the ratio of capillary to elastic force is shown for three different humidity levels. Reprinted with permission from [144], copyright 2011 IEEE.

**Capillary forces:** If a NEM switch is fabricated using wet etching or is operating in humid environments, the capillary forces should be considered. It is increasingly important with down-scaling of a NEM switch, as the ratio of capillary to elastic restoring force of the switching element increases with reducing device dimensions (Figure 15) [144]. In humid environments, the capillary condensation from the surrounding vapour on the NEM switch nanocontacts impacts the adhesion behaviour during switching operation. During a NEM switch fabrication process, the capillary forces may arise from residuals left after lithographic processing using wet etching. The impact of wet fabrication steps can be minimized using super critical point drying [12,162–163]. To prevent the occurrence of capillary forces arising from the device fabrication process, it is recommended to use dry switching element release procedures (e.g., dry etching with HF [5–7], reactive ion etching [5,12,19]).

Methods for minimising/elimination of capillary forces during operation include coating the switch elements with hydrophobic self-assembled monolayers (SAMs) [104,164] and immersing the NEM switch in an insulating liquid environment, which also significantly reduces vdW forces. However, coating with SAMs poses challenges in tailoring the electrical properties of the switch.

**Oxidation:** When designing a NEM switch for operation in ambient environment, the oxidation characteristics of materials must be considered. Regarding electrical contact materials, noble metals are highly resistant to oxidation and corrosion in ambient air at room temperature. However, for Pt-group metals (e.g., Pt, Ir, Ru) the formation of the surface oxide can be activated by the presence of water vapour at relatively low temperatures [165]. In contrast to noble metals, almost all base metals develop a thin oxide layer under ambient conditions. In this case, the chemical and mechanical properties of a native oxide determine the corrosion resistance of NEM switch contact materials. For example, Ni, Cr and Ti form protective layers, making them suitable as electrode coatings in a broad range of environments. The electrical conductivity of metal oxides of contact materials spans a large range of values from metallic to insulating. For example, Ir can form an electrically conductive oxide under proper temperature conditions. Cr oxide is moderately conductive with resistivity in the range of 3 × 10^−6^ Ω·m [166]. Native oxide of Cu is semiconducting (with resistivity around 4.6 × 10^4^ Ω·m at room temperature), showing pronounced temperature dependence [167]. Ni oxidizes to form a crystalline, mechanically robust NiO film at room temperature with a resistivity of approximately 0.5 Ω·m [168]. Ti oxide has high bulk resistivity [169], but for thin films, it can significantly decrease due to the changes in material stoichiometry [170–171]. One of the metal oxides with the highest (5 × 10^11^ Ω·m [172]) is the brittle Al native oxide, growing rapidly under ambient conditions. Commonly used for NEM switching elements, semiconductors Si and Ge in ambient air develop few nanometre thick, insulating (1 × 10^12^ Ω·m (Si) [13,20] and 8 × 10^8^ Ω·m (Ge) [10,173]) oxide layers.

**Figure 16:**
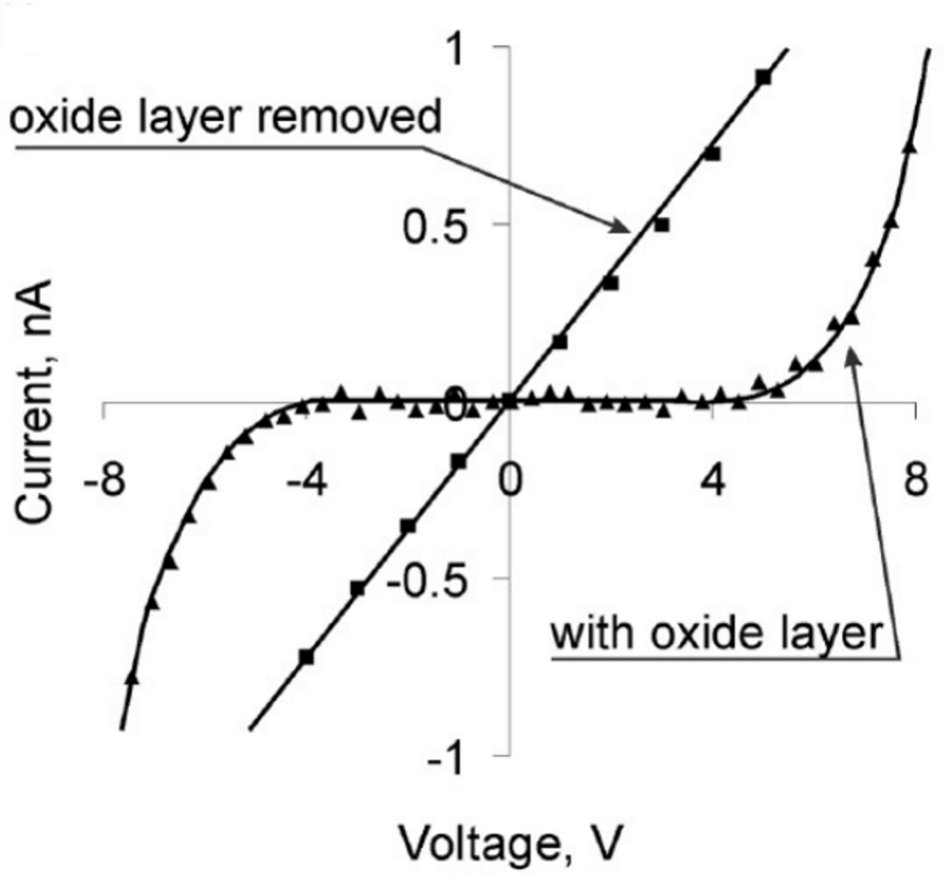
*I*(*V*) characteristics of Ge nanowire with and without an oxide layer. Reprinted with permission from [10], copyright 2009 American Chemical Society.

Typically, the oxidation of NEM switch component materials during fabrication or operation is accompanied by an increase of contact resistance and creation of a potential barrier in the contact [8,13,17,49], manifested in nonlinear *I*(*V*) characteristics (Figure 16). Complete removal of the oxide layer leads to reduction of the potential barrier between the contacting surfaces, resulting in significant improvement of electrical contact and linear *I*(*V*) characteristics, allowing operation at low voltages (Figure 16) [10].

Alternatively, repetitive cycling of a NEM switch may lead to modification (thinning) of the oxide layer in the nanocontact area, reported, for example, during *I*(*V*) cycling of an oxide-covered Ge nanowire–Au nanocontact [54]. Current flow and heating caused by FN tunnelling through the oxide results in its smoothening and thinning in the nanocontact. This leads to a simultaneous increase of the nanocontact area and a decrease of the contact resistance [54].

The electrical charge stored in the poorly conductive oxide layer may enforce the electrostatic attractive force applied to the switching element. A decrease of jump-in voltage during cycling was attributed to the charge build-up effect, reported for NEM switches with Si [20] and Cu [17] switching elements. Also, it has been shown that an oxide layer can significantly alter the Young’s modulus and consequently the stiffness of the switching element [174–175], affecting both jump-in and jump-off voltages of the switch.

**Environmental contaminants:** Ambient hydrocarbons and other organic compounds as traces of organic vapours may form an insulating high molecular weight carbonaceous deposit (tribopolymer) in the areas experiencing mechanical load [158], for instance, during the on switching events. The impact of tribopolymer on NEM switch operation has been experimentally investigated using controlled environmental setups, introducing known levels of organic contaminant gasses such as benzene [156,158]. The proposed explanation of the tribopolymer formation phenomena is adsorption of hydrocarbon molecules on a metal surface followed by chemical interaction between themselves and with the metal surface under mechanical load, becoming polymerized and dehydrogenated [157–158,176]. While a detailed understanding of the tribopolymer formation mechanism is lacking [157,168], it has been related to catalytic activity of contact metals. It has been shown that while Pt group metals are the most susceptible to formation of a tribopolymer layer, it deposits also on the surface of other metals such as Mo, Ta, Cr and Au [157]. The formation of tribopolymer on most metal layers results in significant reduction of adhesion between the contacting materials due to significantly (by approximately three orders of magnitude) decreased surface energy in comparison with pure metals [177]. Calculations have shown that every mechanical switching cycle creates about a monolayer of polymer [157].

The electrical current, flowing through the nanocontact during each switching cycle, has a twofold effect on formation and evolution of the tribopolymer layer. On the one hand, it causes dielectric breakdown of a layer grown during this cycle by permanently altering its structure to a conductive state [158]. On the other hand, similar to mechanical stress, the electrical current increases the rate of polymer growth by supplying additional energy to molecules adsorbed at the metal surface and helps to overcome the activation energy barrier for adsorbent polymerization [156,158]. This process may result in an increase of the electrical resistance of the NEM switch contact by a few orders of magnitude over the contact lifetime of a few billion cycles [63].

#### Operation at extreme temperatures

**Figure 17:**
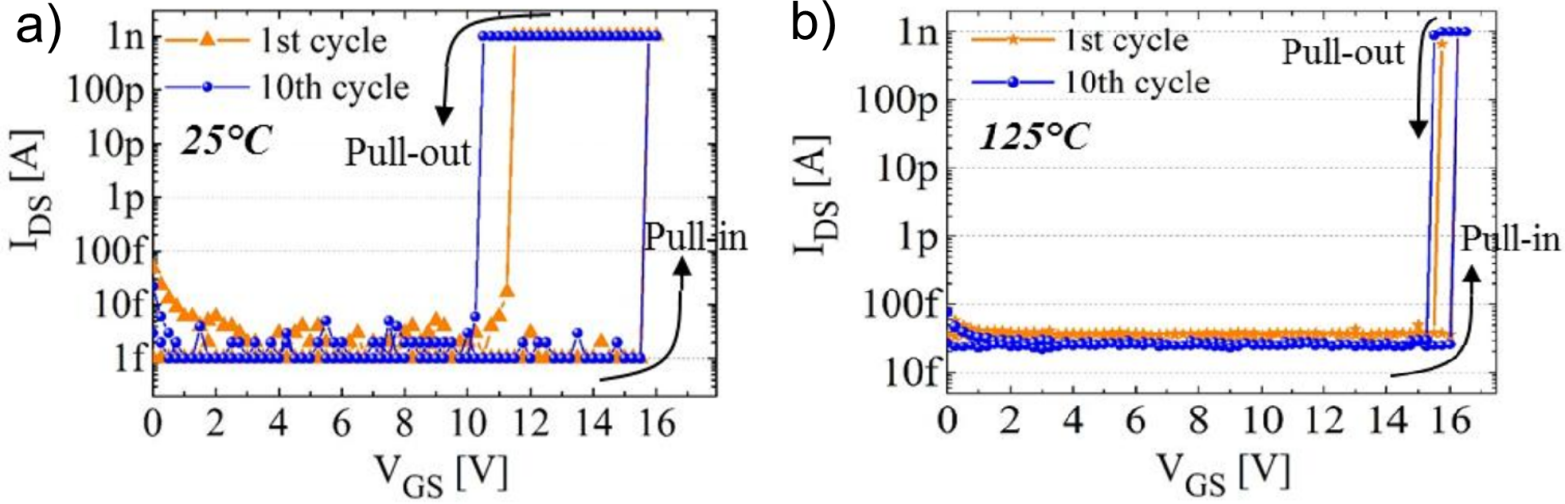
Temperature impact on NEM switch hysteresis. Hysteresis *I*(*V*) loops of a SiGe–TiN NEM relay measured at (a) 25 °C and (b) 125 °C. Reprinted with permission from [6], copyright 2015 IEEE.

One of the strengths of NEM switches are their proposed superiority for harsh environment applications such as extreme temperatures. The operational characteristics at 300 °C for a molybdenum NEM switch and 500 °C for SiC showed that the off-state leakage current is not influenced by temperature [7,19,38]. In comparison with room temperature, operation at higher temperatures has improved the stability of the contact resistance, suggesting that cleaning of the surfaces from moisture and contaminants is taking place [19,158]. Increased temperatures may also release residual stress in the switching element, thus reducing NEM switching hysteresis width (Figure 17) [6]. It is important to note that the temperature impact on Young’s modulus and electrical resistivity of the switching element material needs to be considered when designing the NEM switch. For the majority of semiconductors, both the Young’s modulus and the electrical resistivity significantly decrease with an increase of temperature (references for Si and Ge are given as an example [131,178–179]), while for metals, the high temperature related decrease of the Young’s modulus is accompanied by an increase of electrical resistivity [180]. NEM switch operation has also been demonstrated at low temperatures, for example, for graphene NEM switches at 78 K and 10^−6^ Torr [25].

#### Clean environments

Due to the environmental effects described previously, clean environments such as high vacuum [19,152] or clean N_2_ [149,181–183] are needed to reduce or eliminate oxidation and contamination, as well as to increase the switching speed. To achieve clean operation, sources of environmental contamination (sacrificial layers, organic solvents, exposure to air) must be eliminated during each stage of NEM switch fabrication and testing, including processing, transfer to and testing in the operating environment.

Encapsulation of NEM/MEM devices may be a solution to minimize environmental contamination- and oxidation-caused modification of nanocontacts. However, it increases the device size and the complexity of the fabrication process. Up to now, only a few reports on MEM switch encapsulation attempts can be found [184–185].

### NEM switch failure modes and mechanisms

The following section provides a brief overview of specific nanobeam-based NEM switch related problems that cause a switch to malfunction. The most frequently observed failure modes, their mechanisms, as well as suggestions of possible solutions for preventing these failures are reviewed.

#### Mechanical tear

The estimations show that in NEM contact switches with 2T single-clamped architectures shown in Figure 1a,b, the speed of the free end of the switching element during the accelerated jump-in motion can be as high as 200 m/s [15]. Compressive stress of the switching element at the moment of contacting the drain electrode can reach 30 GPa. It is accompanied by deformation of the switching element, resulting in generation of a strong elastic compression wave propagating along it, and is superimposed on a bending stress in the switching element. This may lead to wear, tear and loss of the contacting material [24,29,186–187], especially over repetitive switching of CNTs used as switching elements [15]. The possible solution for this failure would be the reduction of the separation gap between the contact electrode and the switching element, but that would mean lower retraction forces and higher risk of device failure due to stiction. Another option is to use more durable materials such as semiconductor nanowires [8,10] for the switching elements.

#### Increase of switch resistance resulting in current drop down to the noise level in on state

The switch contact can become electrically insulating due to the oxidation of the switching material or build-up of a non-conductive tribopolymer layer under repetitively applied load in the contact [63,156,188]. Oxidation and tribopolymer formation issues were discussed previously. Both problems can be addressed by encapsulation of the switch in an inert atmosphere, preferably high vacuum, but it increases the device size and complexity of the fabrication process. Alternatively, the use of chemically inert materials or materials covered with electrically conductive oxides may be helpful in minimising these issues. If the dielectric layer has already formed, for example, during the fabrication process or mechanical cycling, it can be demolished by applying higher electrical fields, thus breaking it down [156]. However, this may result in the device failure.

#### Stiction

Permanent stiction occurs when adhesion in the contact between the switching element and the electrode exceeds the pull-out forces, leaving the switching element permanently attached to the contact electrode (on position). The most common ways to prevent stiction of the switching element already during the first switching cycle are: to decrease the contact area [8] (drawback – increased contact resistance and current density through the contact); to increase the initial separation gap between the switching element and the contact electrode [13] (drawback – increased jump-in voltage); to use materials with lower free surface energy [74–76].

During the on–off cycling of a NEM switch, there are several processes (discussed previously), which may eventually result in permanent stiction of the switch such as material transfer, dielectric charging, surface wear and the formation of chemical bonds at the contacting interface.

**Material transfer:** Material transfer caused by thermal evaporation of material from anode electrode (described in detail previously) may be avoided by reducing the source–drain voltage below a critical value [104–105,189]. However, this would increase the risk of stiction for 2T NEM switches due to lower restoring force of the switching element. Also, by increasing the switching speed, the field emission time would be shorter, thus minimising or avoiding the following thermal emission [105,189]. Considering the described material transfer mechanism, the materials with higher thermal conductivity and melting temperature are a preferable choice to resolve this problem.

**Dielectric charging:** Charging of the dielectric material (for example, thin native non-conductive oxide, tribopolymer) covering the contacting surfaces, may force the switch to jump to an on state at voltages lower than the jump-in voltage. For large charge build-up relative to the NEM switch separation gap, this effect may lead to permanent stiction of the device [190]. The charge accumulation issue may be partially resolved by using bipolar AC rather than DC voltage actuation [191]. However, it would require complex electronics [192–193] to drive the NEM switch in dielectric charging-free mode.

**Surface wear:** Surface wear, which develops during repeated on–off cycling of a NEM switch, can lead to an increase of the contact area to the point where stiction occurs. It can be minimised using materials with high hardness and good electrical conductivity, including innovative materials such as Pt*_x_*Si [63]. Soft metals, traditionally used for electrodes, for example, Au, can be strengthened against wear by alloying with other metals [150], however, using of such alloys instead of pure Au may lead to increased current densities in contact. The physical properties of materials which are used in NEM switches were discussed in previous sections.

**Chemical bonds:** The formation of chemical bonds between the contact material and the switching element increases the adhesion forces in the contact. For instance, formation of covalent bonds between Au and S atoms significantly increasing adhesion in the contact, as was reported for a Mo_6_I_3_S_6_-based NEM switch [8]. The process of chemical bond formation may be enhanced by a current-induced temperature increase in the contact area. Most graphene-based devices suffer from irreversible stiction after only a few switching cycles [24–25,27–31,60,80] due to the high current density and corresponding temperature increase in a graphene–gold switch contact. As a result, carbon–gold chemical bonds are established at the interface between the edge of a graphene nanoribbon switching element and a gold contact electrode and leads to permanent stiction of the switch [80].

#### Burn-out

The mechanism of electrical current induced burn-out of a NEM switching element is governed by a set of parameters, which include thermal and electrical conductivities of the switching element, electrode and nanocontact area.

**Figure 18:**
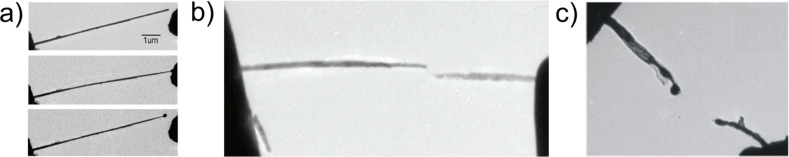
Electrical burn-out induced failure of the NEM switching element. a) Ball formation at the end of a Ge nanowire at the jump-in moment. Reprinted with permission from [10], copyright 2009 American Chemical Society. (b) GaN nanowire and (c) Mo_6_S_3_I_6_ nanowire bundle break in the middle when the voltage is applied in the on state. Reprinted with permission from [96], copyright 2011 American Chemistry Society and from [8], copyright 2010 IOP Publishing.

One of the most common burn-out mechanisms is partial local melting of the electrode material at the contact point and partial ablation of the switching element at the contact. This burn-out mechanism is typical for jump-in events and can be explained by rapid local heating caused by electrostatic discharge at the jump-in-contact moment. In this process, charges accumulated in the off state give rise to a current peak through the contact area that is significantly larger than the steady-state on current [16]. Due to high jump-in voltages, this is the main failure mode of bistable 2T NEM switches. As an example, the burn-out of a Ge nanowire occurred at a jump-in voltage of 13.5 V with current density of 3 nA/nm^2^ in the contact (Figure 18a [10]).

Degradation of a switching element near the contact is also observed for NEM switches in the on state, even if materials with good thermal conductivity are used [10,14].

As the contact area is much smaller than the cross-sectional area of the switching element (see Table 1), the current density in the contact is high enough to cause migration of atoms in the material and lead to failure of the switching element due to large electromigration-induced mechanical stresses [96,99]. When the thermal conductivity of a switching element is relatively low, Joule heating at current densities >0.1 nA/nm^2^ (for GaAs nanowires) and >10 nA/nm^2^ (for Mo_6_S_3_I_6_ nanowires) leads to a high temperature difference between the middle point and the ends and consequent thermal breakdown of the switching element into two halves [8,96] (Figure 18b,c). In Mo_6_S_3_I_6_ nanowires, Joule heating also leads to chemical decomposition and transformation into Mo nanowires through thermal evaporation of S and I atoms. Breakdown of the transformed nanowires occurred at current densities of about 80 nA/nm^2^ [100].

**Figure 19:**
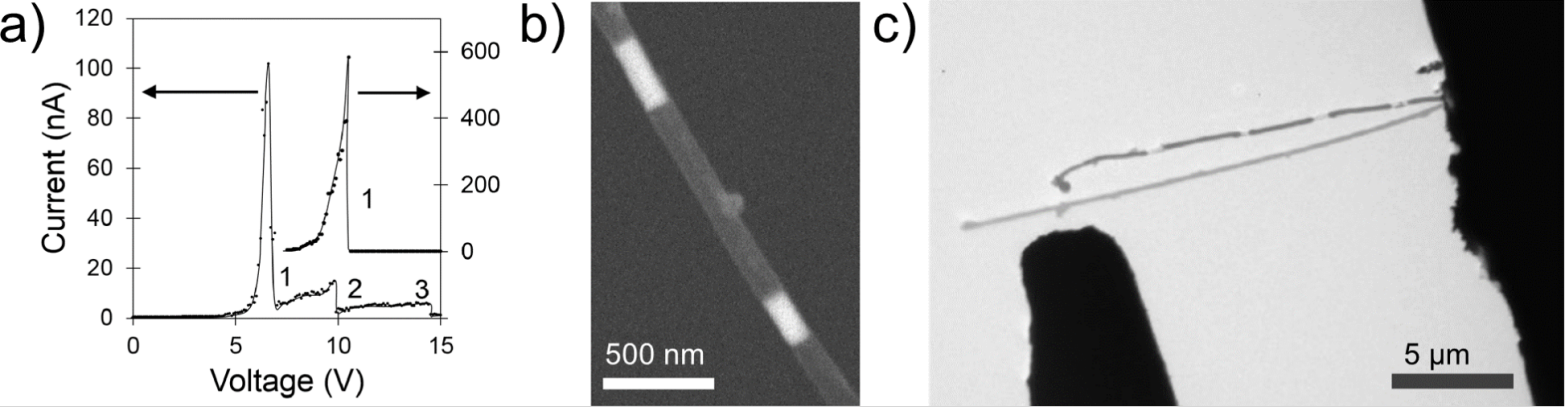
a) Breakdown *I*(*V*) characteristics of two individual Bi_2_Se_3_ nanobelts. 1-2-3 – The step-like breakdown of a nanobelt when the increase of voltage is continued after the first partial breakdown. 1 – single-step complete breakdown of a nanobelt. b) Core meltdown of a Bi_2_Se_3_ nanobelt. c) Core meltdown of a Ge nanowire, captured in both “before” and “after” states of the nanowire in a single frame. Reprinted with permission from [55], copyright 2016 AIPP and from [10], copyright 2009 American Chemical Society.

Native-oxide-coated nanowires may also experience meltdown of the core material at the moment of jump-in contact [10] or during the increase of current densities in on state [55], presumably, due to poor thermal contact between the nanowire and electrode. Figure 19a,b illustrates step-like degradation of a Bi_2_Se_3_ nanowire during the on state. One to several breakdown steps is typical for core/shell nanowires. The first current drop (Figure 19a, marked with 1) occurs when the nanowire core melts and is transformed to a shell-like structure filled with droplets (Figure 19b) [55]. In air ambient Bi_2_Se_3_ nanowires become covered with composite Bi and Se oxide shell [194]. As the melting temperature of Bi_2_Se_3_ (710 °C) is significantly higher than that of SeO_3_ (118 °C) and SeO_2_ (340 °C) and at the same time lower than that of Bi_2_O_3_ (817 °C), it is possible that after the core of the Bi_2_Se_3_ nanowire melts, a further voltage increase leads to conduction through the semiconducting Bi_2_O_3_ shell until the current drops (Figure 19a, marked with 2 and 3), indicating the successive burn-out of the oxide shell.

Germanium nanowire-based NEM switches demonstrated a similar burn-out mechanism for germanium nanowires covered with a native oxide layer [10]. An example of the core–shell burn-out of a native-oxide-covered Ge nanowire at a jump-in voltage of 37 V is shown in Figure 19c [10]. The core of the nanowire melted in the moment of contacting the electrode. As the core of Ge nanowire melted and divided in segments inside Ge oxide tube, the temperature of the nanowire was in the range between 938 and 1115 °C – the melting points of bulk Ge and GeO_2_, respectively. Simulations performed for clarifying the burn-out process showed that when the nanowire in the active contact area is thermally isolated from the electrode, presumably due to the poor mechanical contact, its temperature increases rapidly, reaching the melting point of Ge in 100 ns [10].

Table 4 summarizes the main failure modes of NEM switches and their possible solutions, as well as the affected properties of the NEM switch, in case these solutions are employed.

**Table 4:** Main failure modes of NEM switches.

Failure mode	Reasons	Possible solutions	Affected properties of proposed solutions

mechanical tear [15,24,29,186–187]	high impact speed and following compression stress when active element jumps into the contact	lower switching speed (smaller jump-in voltages), more durable materials	lower jump-in voltages mean weaker retraction force and possible failure due to stiction
increase of switch resistance resulting in current drop in on state down to the noise level [63,156]	oxidation of the contact surfaces and contamination with hydrocarbons when operating in ambient environment	use of chemically inert materials or materials coated with electrically conductive oxides	presence of an oxide layer may lower contact conductivity
		application of higher voltage pulses for dielectric layer breakdown	risk of burn-out failure
		encapsulation	increases the device size and the complexity of the fabrication process
		operation in vacuum	may be insufficient to prevent adsorption of hydrocarbons if the vacuum is not high enough
stiction [24–25,27,31,60,80,104–105,189,193]	adhesion between switching element and contact electrode exceeds restoring (elastic) force of the switching element	decrease of contact area	increase of contact resistance
		increase of initial gap thus increasing the retraction force	increase of jump-in voltage
		use of switching element with high Young’s modulus, thus increasing the retraction force	increase of jump-in voltage
		use of materials with lower surface energy	increase of contact resistance
	material transfer	reduction of source–drain voltage below 5 V	increases risk of stiction in 2T NEM switches due to reduction of restoring force of switching element
		increase of switching speed	high mechanical impact forces
		use of materials with good thermal conductivity, high melting temperature, high work function and low roughness	–
	dielectric charging	use of bipolar AC rather than DC voltage actuation	the charging effect cannot be eliminated completely, more complex electronics required
burn-out [8,10,14,16,55,96,99–100,194]	electrostatic discharge	decrease of jump-in voltage	increases risk of stiction
		use of dielectric layers to increase contact resistance and reduce charge dissipation rate	increase of power dissipation, delay, decrease of noise margin of the device
	Joule heating	use of insulating contact layer and materials with higher melting temperature	insulating layer increases charge buildup and enhances risk of unstable pull-in voltage
		addition of high resistance in series	decreases on/off state current ratio

## Conclusion

This review highlighted the most significant advancements in nanobeam-based electrostatically actuated NEM switch technology that have taken place during the last decade. Progress has been made in various areas relevant for NEM switches, encompassing characterization of single nanostructures and nanocontacts, as well as engineering of different device designs to increase their reliability and longevity.

The field of NEM switch research continues to develop in various directions, exploring new material combinations, testing and fabrication methods, and operating environments. This review considered both the advantages and drawbacks of current nanobeam-based electrostatically actuated NEM switch research and development. For NEM switches to become useful for practical applications, further work should address the remaining challenges, particularly, improvement of reliability and durability of a NEM switch through prevention of its failure during operation. The development of in situ microscopy based methods for real-time monitoring of processes during the device operation are extremely important for designing NEM switches with improved reliability.

Selecting application-specific materials may help to find the best NEM switch design solution. The general materials requirements include high mechanical stiffness (high Young’s modulus), high hardness, low density, low adhesion, low mechanical dissipation, chemical inertness, good electrical and thermal conductivity.

When choosing materials for NEM contact switch application, one should take into account not only the expected operational parameters of the projected NEM contact switch, but also its fabrication approach. Despite the recent improvements in nanofabrication, achieving a high manufacturing yield remains a challenge especially for devices based on materials not suitable for top-down fabrication.

When fabricating the device, the environment in which the device will operate must be taken into account, as the environment may have not only a negative, but also a positive effect on the NEM switch operation. For example, the fabrication of a NEM switch in an oxygen-rich environment may result in the formation of either stable electrically conductive oxides covering the nanostructures, or insulating oxides preventing burn-off of the switching element.
